# CNS Myelin Sheath Lengths Are an Intrinsic Property of Oligodendrocytes

**DOI:** 10.1016/j.cub.2015.07.056

**Published:** 2015-09-21

**Authors:** Marie E. Bechler, Lauren Byrne, Charles ffrench-Constant

**Affiliations:** 1 MRC Centre for Regenerative Medicine, The University of Edinburgh, 5 Little France Drive, Edinburgh EH16 4UU, UK

## Abstract

Since Río-Hortega’s description of oligodendrocyte morphologies nearly a century ago, many studies have observed myelin sheath-length diversity between CNS regions [1–3]. Myelin sheath length directly impacts axonal conduction velocity by influencing the spacing between nodes of Ranvier. Such differences likely affect neural signal coordination and synchronization [4]. What accounts for regional differences in myelin sheath lengths is unknown; are myelin sheath lengths determined solely by axons or do intrinsic properties of different oligodendrocyte precursor cell populations affect length? The prevailing view is that axons provide molecular cues necessary for oligodendrocyte myelination and appropriate sheath lengths. This view is based upon the observation that axon diameters correlate with myelin sheath length [1, 5, 6], as well as reports that PNS axonal neuregulin-1 type III regulates the initiation and properties of Schwann cell myelin sheaths [7, 8]. However, in the CNS, no such instructive molecules have been shown to be required, and increasing in vitro evidence supports an oligodendrocyte-driven, neuron-independent ability to differentiate and form initial sheaths [9–12]. We test this alternative signal-independent hypothesis—that variation in internode lengths reflects regional oligodendrocyte-intrinsic properties. Using microfibers, we find that oligodendrocytes have a remarkable ability to self-regulate the formation of compact, multilamellar myelin and generate sheaths of physiological length. Our results show that oligodendrocytes respond to fiber diameters and that spinal cord oligodendrocytes generate longer sheaths than cortical oligodendrocytes on fibers, co-cultures, and explants, revealing that oligodendrocytes have regional identity and generate different sheath lengths that mirror internodes in vivo.

## Highlights

• Oligodendrocytes, not Schwann cells, form myelin sheaths without axonal signals
• Oligodendrocytes sense diameter, increasing sheath length with larger fibers
• Oligodendrocytes from cortex and spinal cord form sheaths of different lengths
• Regional differences in sheath lengths may initially be hard-wired

## Graphical Abstract

**Figure:**
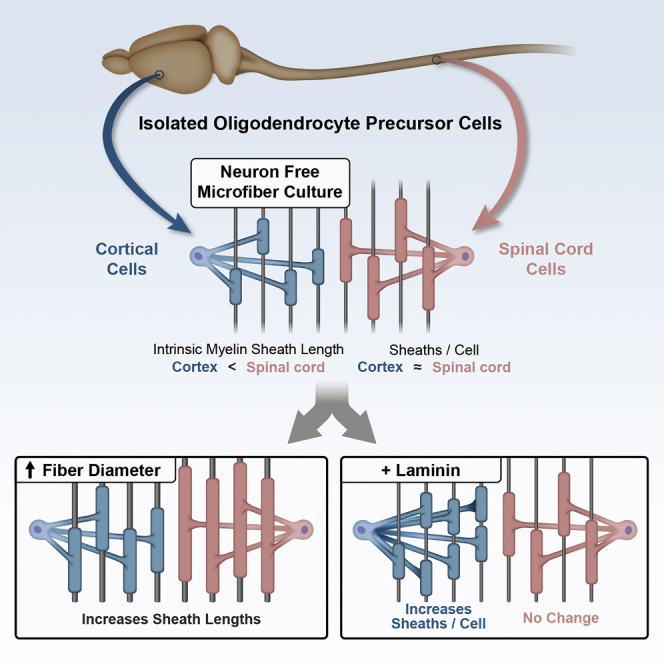

## Teaser

Much of the mammalian brain comprises axons wrapped by myelin sheaths, whose length determines conduction velocity. Bechler et al. overturn the long-held view that sheath-forming oligodendrocytes are all the same. Oligodendrocytes from different regions generate sheath lengths on microfibers and neurons that reflect their in vivo origin.

## Results

**Figure 1:**
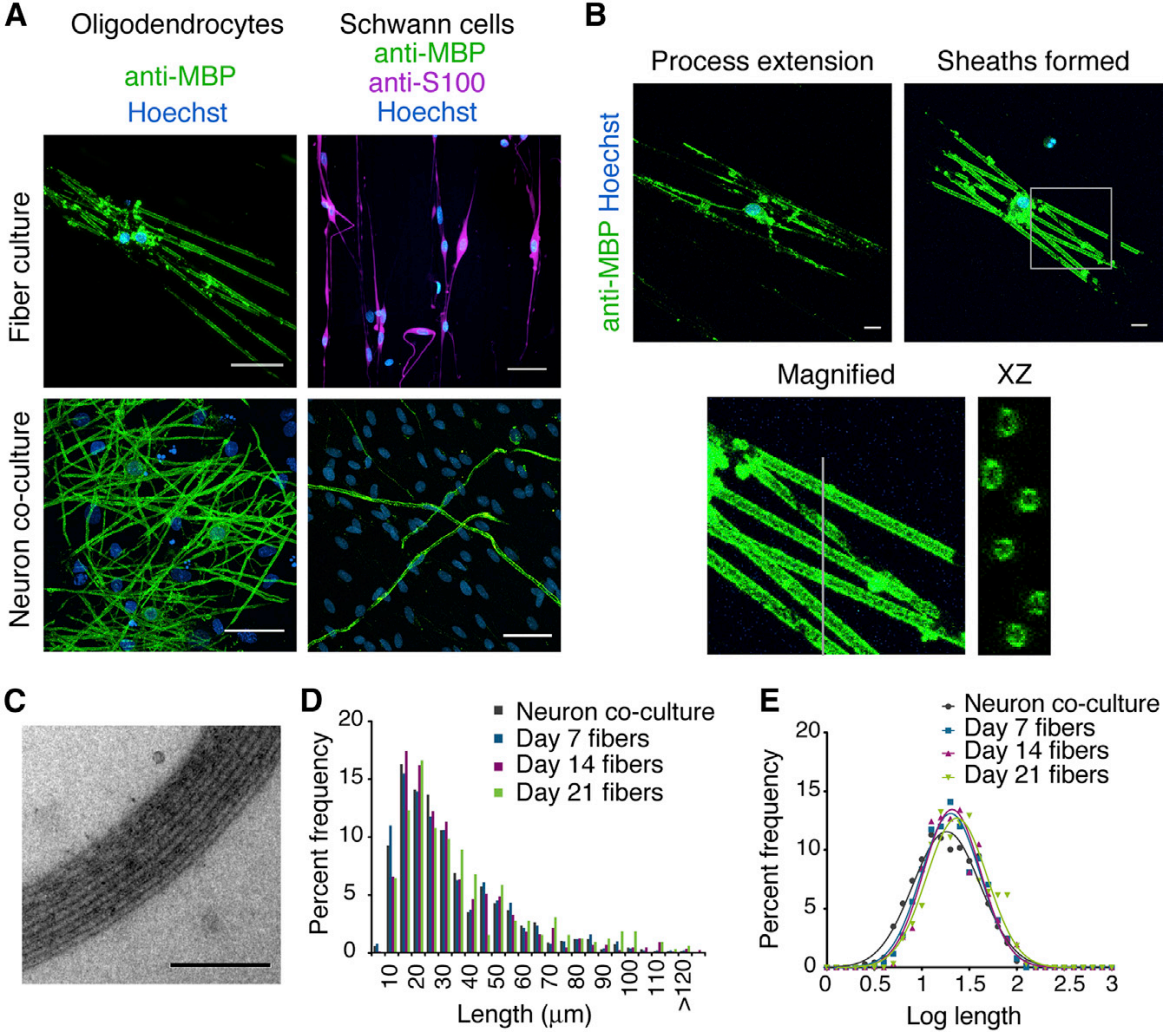
Oligodendrocytes Have the Unique, Intrinsic Capability to Generate Compact Membrane Sheaths and Physiological Internode Lengths on Microfibers (A) Confocal stacks of rat primary cortical oligodendrocytes or Schwann cells cultured 14 or 21 days, respectively, on 1–2 μm microfibers or neurons. The scale bars represent 40 μm. (B) Representative confocal images showing the distinction between process extension and sheath formation. Magnified and cross-section (xz) images are shown on the bottom. The scale bars represent 10 μm. (C) Electron micrographs of multi-layered oligodendrocyte membranes around microfibers by 14 days. The scale bar represents 200 nm. (D) Sheath length histogram showing percent frequency in 5 μm bins. More than 700 sheaths were measured from three experiments, each with pooled cells from greater than six animals. (E) Log transformation of lengths shows Gaussian distributions with no significant difference in mean (one-way ANOVA). See also Figure S1 and Table S1.

In order to distinguish oligodendrocyte-driven versus axon-instructed myelin sheath formation, we established a neuron-free, three-dimensional culture system with poly-L-lactic acid (PLA) microfibers (Figure S1A). Most cortical oligodendrocyte precursor cells differentiated into oligodendrocytes, as seen by myelin basic protein (MBP) expression and a corresponding reduction in NG2 at 7 days (Figure S1B). These oligodendrocytes formed MBP^+^ sheaths surrounding microfibers (Figures 1A and 1B), as in previous reports [12], with 90% ± 4% of MBP^+^ cells ensheathing by 14 days. We verified that sheaths formed on fibers contain other late oligodendrocyte markers, such as myelin oligodendrocyte glycoprotein (MOG), by immunolabeling (Figure S1C). We also confirmed that they formed multi-layered compacted membranes characteristic of myelin sheaths by electron microscopy (Figure 1C), with the majority of processes contacting fibers generating sheaths of two to ten layers (Figure S1D). Whereas both Schwann cells and oligodendrocytes myelinated dorsal root ganglia (DRG) neurons (Figure 1A), Schwann cells did not differentiate or wrap the microfibers when cultured under identical conditions (Figure 1A), showing a fundamental difference in their requirements for axonal signals in myelination. Next, we examined whether isolated cortical oligodendrocytes produce myelin sheath lengths equivalent to that found on axons. MBP^+^ sheaths were measured from oligodendrocyte cultures with DRG neurons or comparable caliber (1–2 μm) microfibers [13]. Within 7 days, sheath lengths were identical to those seen in co-culture with DRG neurons (Figures 1D and 1E; Table S1) and were comparable to reported in vivo cortex distributions [14].

**Figure 2:**
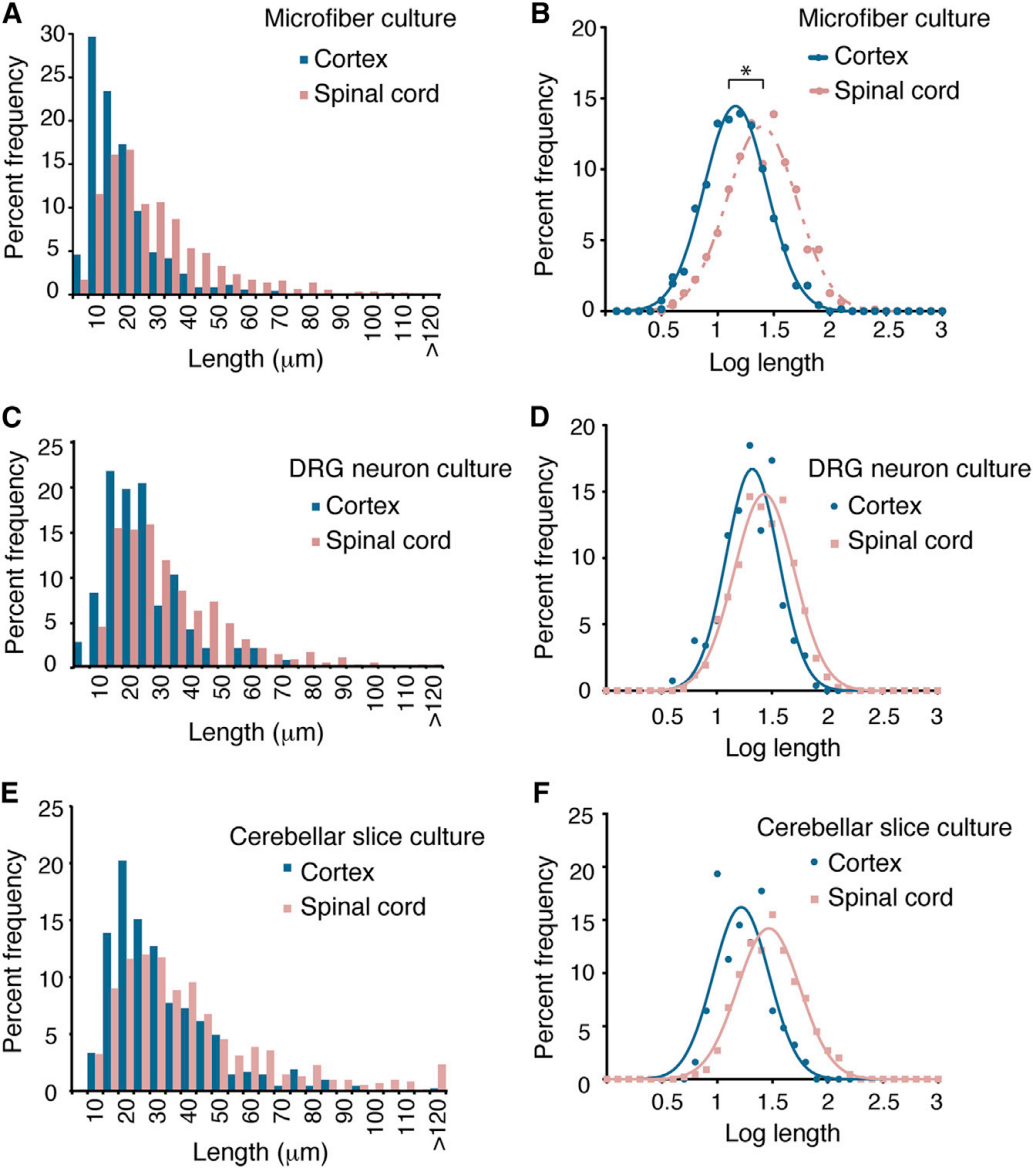
Oligodendrocytes Have Regional Identity that Determines Relative Internode Lengths (A) Histogram of sheath lengths formed by spinal cord- or cortex-isolated oligodendrocytes cultured 14 days on 1–2 μm microfibers. (B) Log sheath lengths on 1–2 μm fibers. ^∗^ indicates p < 0.01; two-tailed t test of mean log lengths. More than 700 sheaths were measured from five experiments, each with pooled cells from greater than three animals. (C) Histogram of sheath lengths formed by either spinal cord- or cortex-isolated oligodendrocytes cultured 14 days on DRG neurons. For each region, more than 250 sheaths were measured from two DRG cultures with a pool of cells from greater than five animals. (D) Log sheath length plot for cultures on DRG neurons. (E) Histogram of sheath lengths formed by either EGFP-expressing spinal cord or cortical oligodendrocytes added to *shiverer* mouse cerebellar slice cultures for 14 days. For each region, more than 400 sheaths were measured from two slices with a pool of cells from greater than five animals. (F) Log sheath length plot for oligodendrocytes cultured on *shiverer* cerebellar slices. See also Figure S2 and Table S2.

In vivo, spinal cord internodes are on average twice the length of cortical internodes [1, 15]. To determine whether oligodendrocytes possess regional identity that governs the lengths of sheaths, we examined oligodendrocyte precursor cells from spinal cord and cortex in the absence of environmental variation, by culturing cells under identical conditions on 1–2 μm microfibers. Oligodendrocyte precursor cells isolated from spinal cord showed similar purity, density, differentiation, and ensheathment on microfibers to cortical oligodendrocytes (Figures S2A–S2D). Surprisingly though, the sheath lengths formed by spinal cord oligodendrocytes were significantly longer than cortical oligodendrocytes (Figures 2A, 2B, and S2F; Table S2). By contrast, the number of sheaths formed was not significantly different between the two populations (Figure S2E). This shows for the first time that oligodendrocytes from two CNS regions are not the same but instead have intrinsic differences that dictate relative sheath lengths.

We confirmed that the longer sheaths in the neonatal spinal cord cells did not result from the differences in the timing of oligodendrocyte development in the two tissues (as myelination starts earlier in the spinal cord than in the cortex) by repeating the analysis using spinal cord oligodendrocyte precursors at E15.5 prior to the onset of myelination in this region. Once again, these cells formed longer sheaths (Figures S2G–S2I). We also confirmed the oligodendrocyte-intrinsic nature of sheath lengths in three ways. First, we mixed cortical and spinal cord oligodendrocyte precursor cells in a single culture, using lentiviral expression of EGFP to distinguish the two populations (Figures S2J and S2K). Second, we cultured the cortical and spinal cord oligodendrocyte precursor cells on DRG neurons (Figures 2C and 2D). Third, we added the two populations to cerebellar slices of *shiverer* mutant mice that lack MBP, enabling the MBP^+^ myelin sheaths formed by the added cells to be visualized and measured within CNS tissue (Figures 2E, 2F, and S2L; Table S2). In all cases, the spinal cord cells generated longer sheaths. Together, these show that external signals from other CNS cell types do not contribute to the signaling responsible for the relative differences in sheath length.

**Figure 3:**
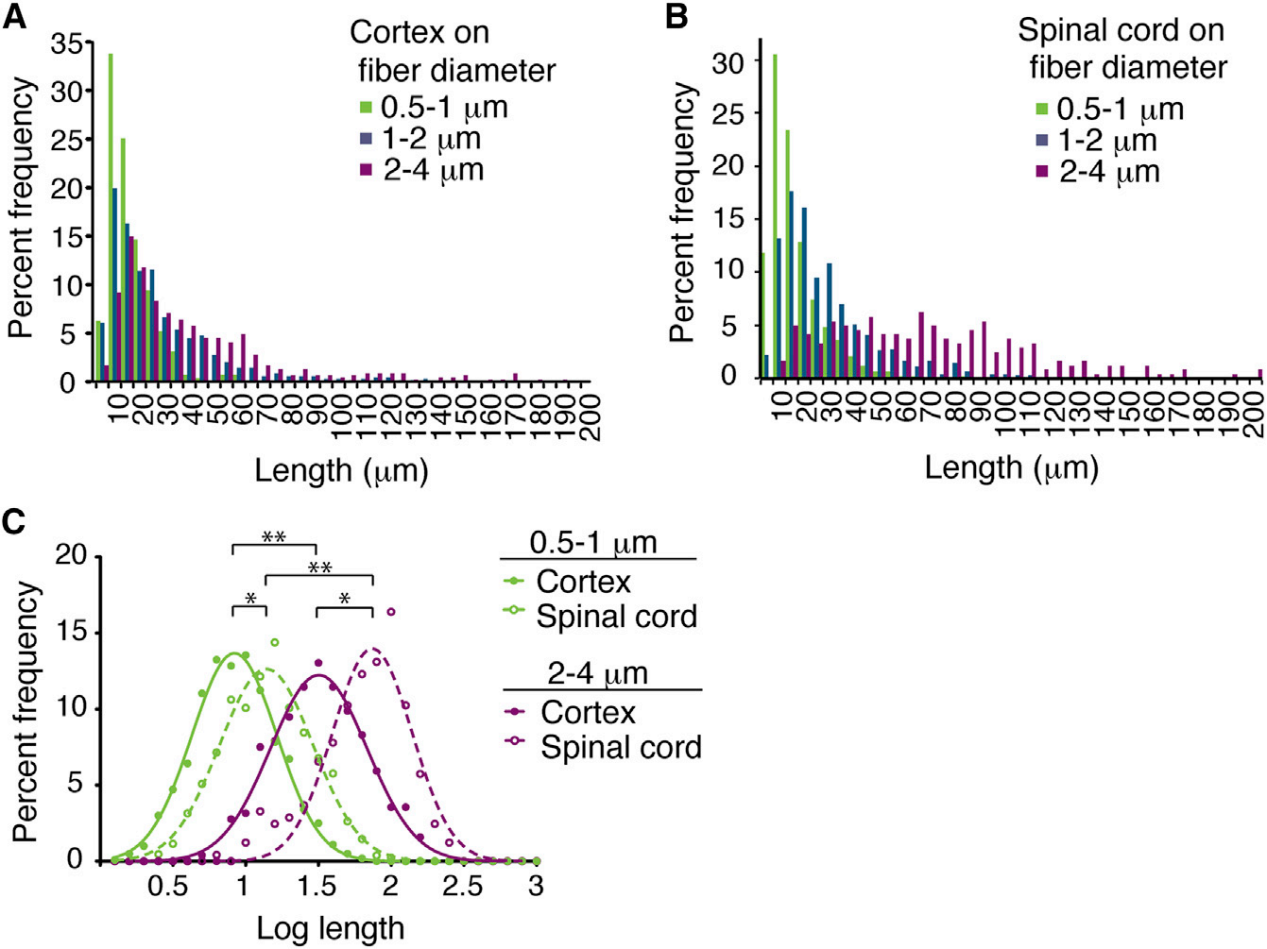
Regional Identity and Physical Cues Explain Differences in Internode Lengths between Cortex and Spinal Cord (A) Sheath lengths of cortical oligodendrocytes increase with increased diameter microfibers. (B) Spinal cord oligodendrocyte sheath lengths also increase with increased diameter microfibers. (C) Log sheath lengths show significant differences between spinal cord and cortical oligodendrocytes on all diameter ranges of microfibers (^∗^ indicates p < 0.03; two-tailed t test for mean log lengths) and across microfiber diameters for cells from the same region (^∗∗^ indicates p < 0.01; one-way ANOVA for mean log lengths). More than 250 sheaths were measured from four experiments, each with pooled cells from greater than three animals. See also Figure S3 and Table S3.

As oligodendrocytes generally form longer internodes on larger-diameter axons in vivo and axon calibers are larger in spinal cord [1], we asked whether oligodendrocytes recognize different caliber axons to generate appropriate-length myelin sheaths in the absence of axonal molecules. To assess the ability of oligodendrocyte processes to respond to purely physical cues, we cultured oligodendrocytes on microfibers of varied diameter ranges: 0.5–1 μm; 1–2 μm; and 2–4 μm (Figures S1A and S3A). The percent of cells expressing MBP was not affected (Figure S3B). The number of sheaths formed was slightly reduced on the smallest-caliber microfibers but did not differ significantly on fibers greater than one micron in diameter (Figure S3C). Cortical oligodendrocytes cultured 14 days on microfibers demonstrated a significant increase in sheath lengths on larger-diameter microfibers (Figures 3A and S3A; Table S3), showing a remarkable ability for oligodendrocytes to respond to caliber by changing sheath lengths. Similarly, spinal cord oligodendrocytes had longer sheath lengths on larger microfiber diameters (Figure 3B; Table S3). Importantly, longer spinal cord sheath lengths, relative to cortex, were found on all microfiber diameters (Figures 3A–3C), demonstrating that, in addition to any effect of the larger-diameter axons in spinal cord, intrinsic differences in the control of cell shape between oligodendrocyte populations result in internode length differences.

**Figure 4:**
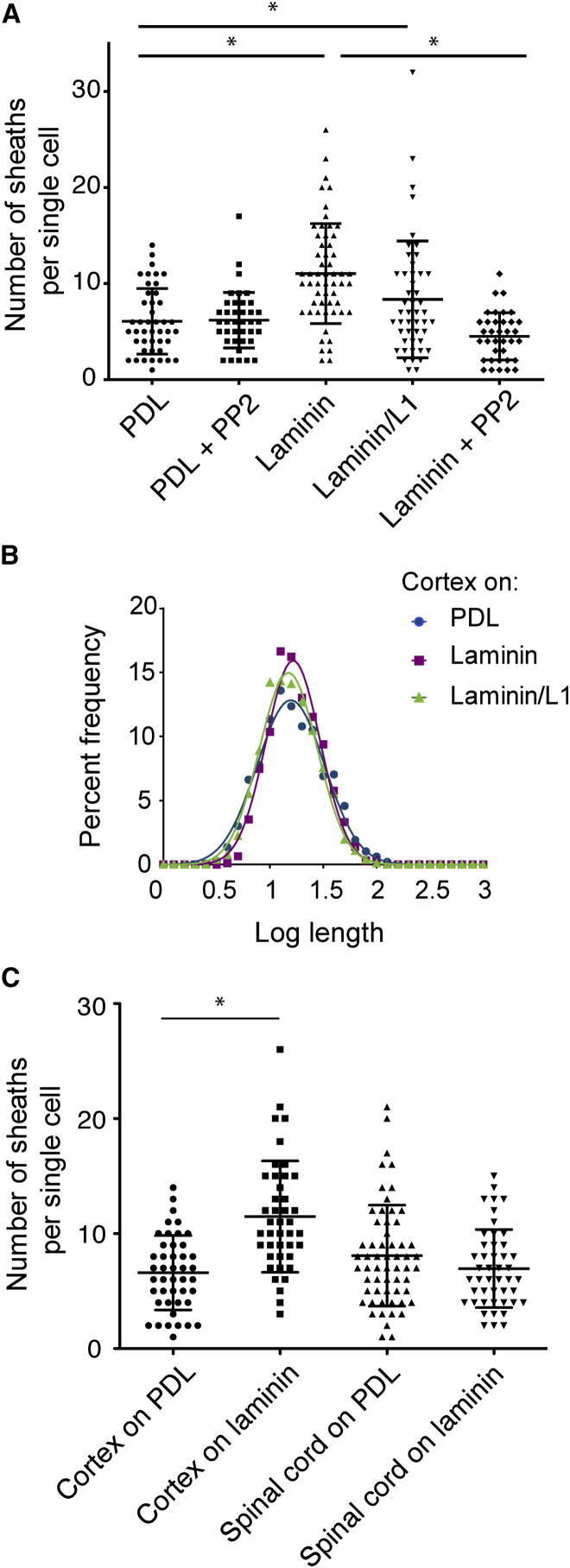
Cortical, but Not Spinal Cord, Oligodendrocytes Respond to Laminin by Producing More Sheaths per Cell (A) Laminin, dependent on Fyn activity, increases the mean sheaths per single cortical oligodendrocyte 2-fold. Cortical oligodendrocytes were cultured 14 days on poly-D-lysine (PDL)-coated 1–2 μm microfibers with or without laminin or laminin + L1 coating and addition of the Fyn inhibitor PP2. Sheath number was analyzed for individual cells. Average with SD is indicated by bars. ^∗^ indicates p < 0.01, Kruskal-Wallis with Dunn’s post-test, with a minimum 42 single cells analyzed for each condition from three experiments, each with pooled cells from greater than three animals. (B) Log transformation of cortical oligodendrocyte sheath length shows no significant difference in the mean (one-way ANOVA) in the presence of laminin. (C) The number of sheaths per individual spinal cord oligodendrocyte does not increase in the presence of laminin. At least 42 individual cells were analyzed from three experiments, each with pooled cells from greater than three animals. See also Figure S4 and Table S4.

Several axonal signals, such as laminin2, have been shown to modulate myelin sheath formation [16]. We asked whether axonal molecules known to enhance myelination modulate the oligodendrocyte-intrinsic program controlling myelin sheath lengths and whether there are region-specific responses. Axonal laminin2 and neuronal cell adhesion molecule L1 have been shown to enhance the formation of myelin sheaths in cortical oligodendrocytes through activation of the Src family kinase Fyn [16]. Therefore, we examined whether PLA microfibers coated with laminin with and without L1 affect sheath formation. One to two micron microfibers were coated with poly-D-lysine (PDL) with or without laminin and L1 (Figure S4A). No significant effect on differentiation was found (cells expressing MBP; Figure S4B). Cortical oligodendrocyte sheath lengths were also unaffected by the addition of laminin or laminin in combination with L1 (Figures 4B, S4C, and S4D; Table S4). However, laminin coating increased the number of sheaths per cortical oligodendrocyte, which was not seen in the presence of the Fyn inhibitor PP2 (Figure 4A). This increase in sheath number, but not length, is consistent with our previous results with activated Fyn expression in zebrafish oligodendrocytes [17]. To address whether oligodendrocytes from the cortex and spinal cord respond similarly to laminin, we cultured spinal cord or cortical oligodendrocytes on PDL- or PDL and laminin-coated microfibers. Unlike cortical oligodendrocytes, the number of sheaths formed by individual spinal cord oligodendrocytes was not increased with laminin-coated microfibers (Figure 4C). These results further highlight regional differences between cortical and spinal cord oligodendrocytes, demonstrating divergent responses to molecules that modulate myelin sheath formation.

## Discussion

Using a microfiber culture system in which oligodendrocytes ensheath inert fibers [12], we find that these myelinating cells of the CNS have a remarkable intrinsic capability to form compact sheaths of expected lengths without molecular instruction from axons. We further find that oligodendrocyte precursor cells are programmed to generate sheath lengths that reflect their in vivo origin, demonstrating that oligodendrocyte precursor cells have acquired a regional identity prior to differentiation and that this identity instructs the signaling pathways that determine sheath length. Together, these intrinsic properties would, without any signals from axonally derived molecules, generate a diversity of oligodendrocyte morphologies within the CNS that has been documented for nearly a century without any understanding of mechanism.

What then is the role of the axon in regulating myelination? We suggest that, contrary to the long-held belief that axonal signals actively direct (and are required for) the process of myelin sheath formation, they are rather responsible for modifying a “hard-wired” pattern established by the intrinsic properties above. Our work shows two mechanisms by which this adaption might be achieved: first by axon size, with oligodendrocytes capable of responding to increased fiber caliber by increasing sheath length, and second by axonal molecules that adapt thickness or sheath number. Myelin thickness has been shown to change with altered levels of neuregulin-1 type III [18, 19]. The number of sheaths formed by oligodendrocytes can be increased by activating Fyn signaling [17], and here, we find that laminin-activated Fyn signaling increases sheath number, but not lengths, in cortical oligodendrocytes. The observation of greater variance of sheath lengths in the cerebellar slice experiments (shown in Figure 2E) points to a role for further signals from an in vivo milieu. The signaling mechanisms by which the oligodendrocytes themselves regulate sheath length remain unknown, and the link between these pathways, a mechanical signal sensitive to axon diameter as illustrated by our results, and other in vivo signals will be important points for further work.

Importantly, these signaling pathways that affect sheath properties may differ in regions of the CNS. Not only do developmentally different sources of oligodendrocyte precursors [20, 21] show regional identity reflected by sheath lengths, we find the response to the environmental cue laminin is also region specific. Our result that cortical, but not spinal cord, oligodendrocytes increase sheath number in response to laminin leads to the conclusion that integrin-activated Fyn signaling is not instructive for myelination in spinal cord oligodendrocytes. This conclusion is consistent with the observations that spinal cord myelination is normal in Fyn KO mice whereas myelin in the forebrain is diminished and that mice lacking laminin α2 have a decrease in myelinated axons in the brain but show very little effect for spinal cord myelination [22, 23].

An intrinsic pathway that sets up a pre-determined pattern of myelination followed by extrinsic cues that then modify the myelin sheaths points to a flexible developmental mechanism. Such a mechanism would establish an initial network capable of rapid neuronal conduction that can then be sculpted by environmental signals. Our demonstration that sheath length and number are regulated independently (as evidenced by the effects of axon size on sheath length and laminin on sheath number), together with the work of others showing that neuronal activity can affect myelin thickness [24–26], emphasizes the potential adaptability of the cortical oligodendrocytes in response to these environmental signals. Together with the demonstration of an intrinsic program to establish a basic pattern of myelination, it suggests a sequence analogous to synaptogenesis in the CNS, in which a hard-wired pattern established during development is adapted by experience-dependent activity. Each of the adaptable variables (length, number, and thickness) will regulate conduction velocities along single axons, and it will be important in future work to establish, by a combination of experimental approaches and computational modeling, the extent to which adaptive myelination occurs and could contribute to the plasticity of the CNS that underpins higher cognitive functions such as learning.

## Supplemental Information

Document S1. Supplemental Experimental Procedures, Figures S1–S4, and Tables S1–S4

Document S2. Article plus Supplemental Information
