# Supplementary material for: CNS Myelin Sheath Lengths Are an Intrinsic Property of Oligodendrocytes
Source: Curr Biol. 2015 Sep 21;25(18):2411–6. doi: 10.1016/j.cub.2015.07.056 (PMC4580335; doi:10.1016/j.cub.2015.07.056)
Supplement: Document S1. Supplemental Experimental Procedures, Figures S1–S4, and Tables S1–S4 [file mmc1.pdf]

Current Biology

Supplemental Information

CNS Myelin Sheath Lengths

Are an Intrinsic Property of Oligodendrocytes

Marie E. Bechler, Lauren Byrne, and Charles ffrench-Constant

Supplemental Data

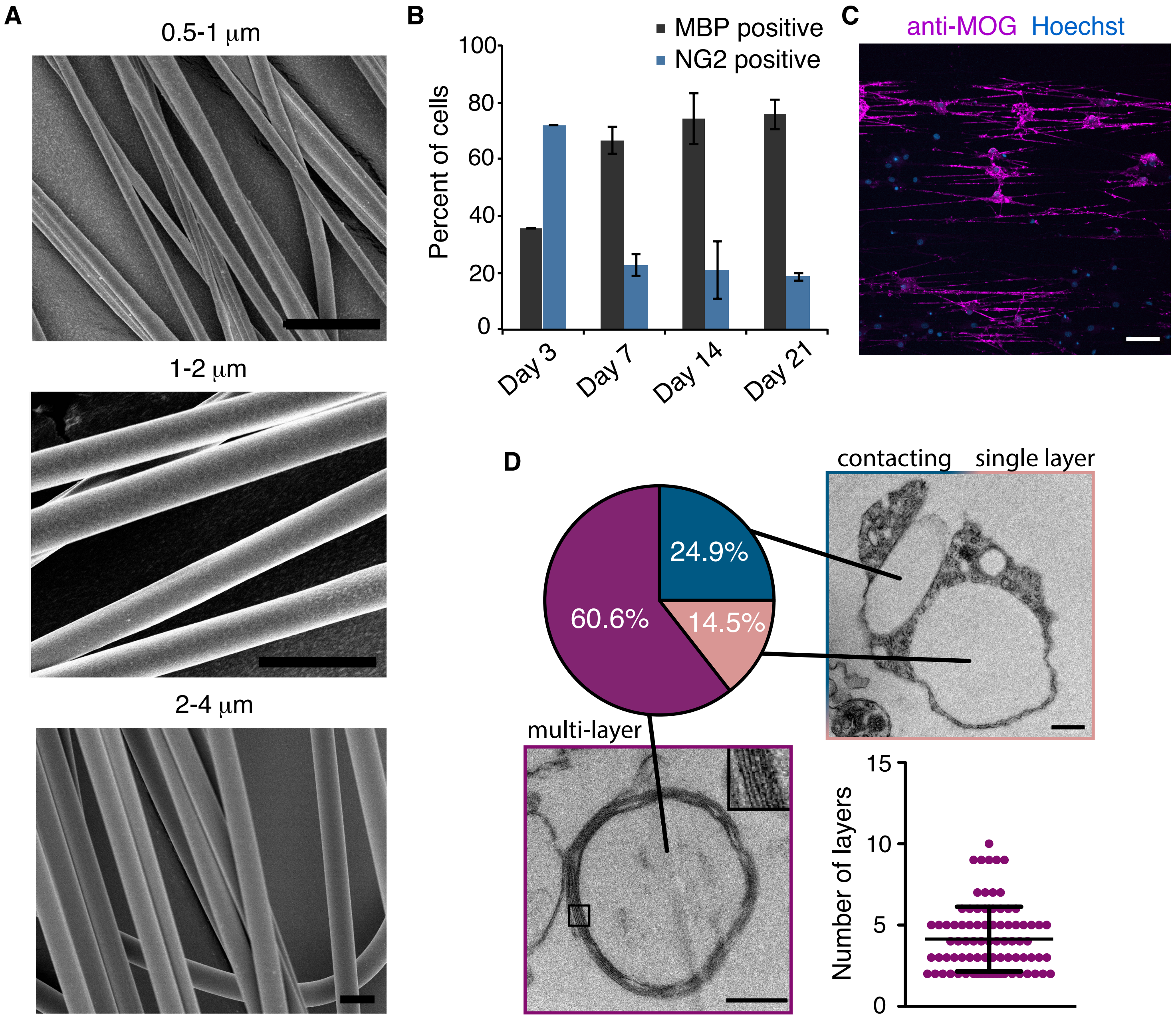

Figure S1. Oligodendrocyte precursors differentiate to mature oligodendrocytes on microfibers. Also see Figure 1. **(A)** Scanning electron micrographs of 0.5-1 µm, 1-2 µm, or 2-4 µm diameter fibers. Scale bars, 5 µm. **(B)** Percent of oligodendrocyte precursors (NG2) and oligodendrocytes (MBP) on 1-2 µm diameter microfibers. More than 600 cells were counted per condition. Mean and standard deviation are shown for three experiments, each with pooled cells from >3 animals. **(C)** Confocal stack shows that mature oligodendrocyte protein MOG is expressed and localized in sheaths. Scale bar, 40 µm. **(D)** Pie chart shows the percent of oligodendrocyte-fiber contact sites categorized as contacting only, single layer wrapping, and multi-layer wrapping. Example images of categories are shown. The number of layers of multilamellar wrapping is shown.

Table S1. Average sheath lengths with standard deviation.

The average log length and standard deviation were used for statistical tests, as the sheath length data was log normal (log lengths were Gaussian distributions). The equivalent mean sheath length in microns ± one standard deviation is shown (as lower and upper sheath length), calculated from the mean log(length) ± standard deviation of log(length).

Related figure	Condition	Mean log(length)	Standard deviation log(length)	Mean length (microns)	Lower length (microns)	Upper length (microns)
1 D,E	Day 21 neuron culture	1.300	0.022	20.0	19.0	21.0
1 D,E	Day 7 fiber culture	1.377	0.054	23.8	21.0	27.0
1 D,E	Day 14 fiber culture	1.370	0.100	23.4	18.6	29.5
1 D,E	Day 21 fiber culture	1.415	0.087	26.0	21.3	31.7

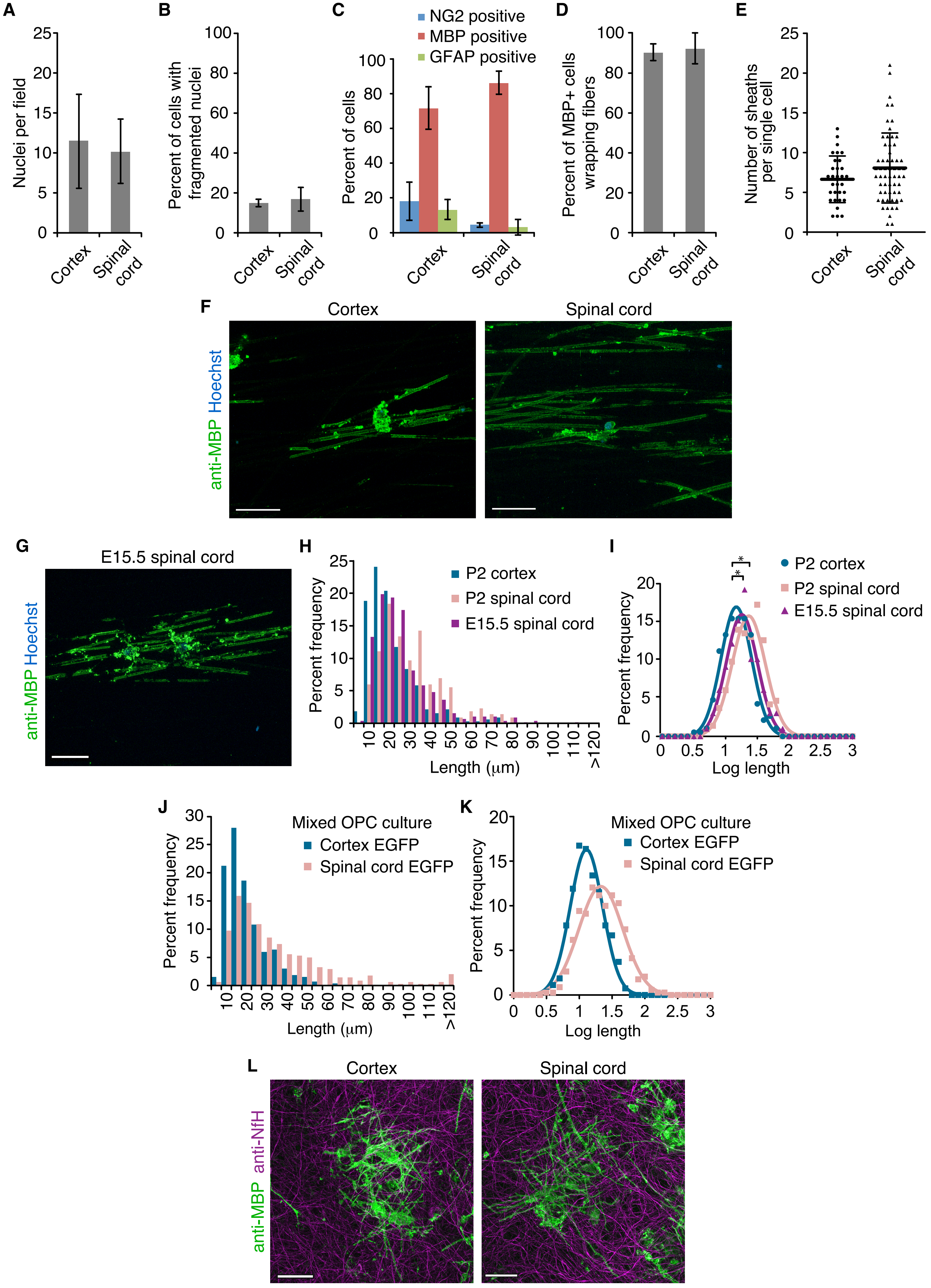
A
Nuclei per field
Cortex
Spinal cord
B
Percent of cells with fragmented nuclei
Cortex
Spinal cord
C
NG2 positive
MBP positive
GFAP positive
Percent of cells
Cortex
Spinal cord
D
Percent of MBP+ cells wrapping fibers
Cortex
Spinal cord
E
Number of sheaths per single cell
Cortex
Spinal cord
F
Cortex
Spinal cord
anti-MBP Hoechst
G
E15.5 spinal cord
anti-MBP Hoechst
H
P2 cortex
P2 spinal cord
E15.5 spinal cord
Percent frequency
Length (μm)
I
P2 cortex
P2 spinal cord
E15.5 spinal cord
Percent frequency
Log length
J
Mixed OPC culture
Cortex EGFP
Spinal cord EGFP
Percent frequency
Length (μm)
K
Mixed OPC culture
Cortex EGFP
Spinal cord EGFP
Percent frequency
Log length
L
Cortex
Spinal cord
anti-MBP anti-NfH

Figure S2. Oligodendrocytes have intrinsic regional identity that determines relative sheath lengths. Also see Figure 2. **(A)** The cell density of cortical and spinal cord oligodendrocytes is comparable after 14 days in culture on 1-2 μm microfibers, as seen by number of nuclei per image field. More than 500 cells were counted per condition. Mean and standard deviation are shown for five experiments, each with pooled cells from >3 animals. **(B)** A similar fraction of cells undergo cell death between cortical and spinal cord oligodendrocytes, as determined by fragmented nuclei after 14 days on 1-2 μm microfibers. More than 350 cells were counted per condition. Mean with standard deviation is shown for three experiments, each with pooled cells from >3 animals. **(C)** The percent of oligodendrocyte precursor cells (NG2 positive), differentiated oligodendrocytes (MBP positive), and astrocytes (GFAP positive) between cortex and spinal cord preparations is shown for Day 14 on 1-2 μm microfibers. More than 300 cells were counted per condition. Mean with standard deviation is shown for three experiments, each with pooled cells from >3 pooled animals. **(D)** The percent of MBP+ cells generating at least one sheath is shown for Day 14 spinal cord and cortical oligodendrocyte cultures on 1-2 μm fibers. **(E)** The number of sheaths formed by individual spinal cord or cortical oligodendrocytes is shown with mean and standard deviation for at least 31 cells from four experiments, each with pooled cells from >3 animals. **(F)** Confocal stacks of rat primary oligodendrocytes isolated from neonatal cortex or spinal cord, cultured 14 days on 1-2 μm microfibers. Scale bars, 40 μm. **(G)** Example of a confocal stacked image of E15.5 spinal cord oligodendrocytes on 1-2 μm microfibers cultured 14 days. Scale bar, 40 μm.
(H) Histogram of sheath lengths at Day 14 on 1-2 μm microfibers. More than 200 sheaths per condition were measured from 3 experiments, each with pooled cells from >3 animals. **(I)** Log sheath length frequency of neonatal cortical or spinal cord oligodendrocytes versus E15.5 spinal

cord oligodendrocytes is shown. Spinal cord oligodendrocyte (E15.5 or neonatal) mean log sheath lengths are significantly different than cortex, * $p < 0.02$, one-way ANOVA for mean log lengths. (**J**) Histogram showing sheath lengths from lentivirus-transduced, EGFP-expressing oligodendrocytes. EGFP-expressing spinal cord oligodendrocytes were mixed with unlabeled cortical oligodendrocytes, or EGFP-expressing cortical oligodendrocytes with unlabeled spinal cord oligodendrocytes, verifying that the sheath length differences are intrinsic to oligodendrocytes from these two regions. More than 250 sheaths were measured from two experiments, each with pooled cells from >6 pups added. **(K)** Frequency of log sheath lengths from indicated EGFP-expressing oligodendrocyte populations in mixed oligodendrocyte cultures. **(L)** Example confocal images of cortical and spinal cord oligodendrocytes added to cerebellar *shiverer* slice cultures. Scale bar, 40 μm.

Table S2. Average sheath lengths with standard deviation.

The average log length and standard deviation were used for statistical tests. The equivalent mean sheath length with lower and upper sheath lengths (mean + or – one standard deviation) is shown, calculated from the mean log(length) ± standard deviation of log(length).

Related figure	Condition	Mean log length	Standard deviation log length	Mean length (microns)	Lower length (microns)	Upper length (microns)
2 A,B	Cortex on fibers	1.091	0.150	12.3	8.7	17.4
2 A,B	Spinal cord on fibers	1.353	0.061	22.5	19.6	25.9
2 C,D	Cortex on neurons	1.403	0.100	25.3	20.1	31.8
2 C,D	Spinal cord on neurons	1.465	0.116	29.2	22.3	38.1
2 E,F	Cortex on slices	1.347	0.092	22.2	18.0	27.5
2 E,F	Spinal cord on slices	1.471	0.087	29.6	24.2	36.1
S2 G-I	P2 cortex	1.194	0.074	15.6	13.2	18.5
S2 G-I	P2 spinal cord	1.384	0.033	24.2	22.4	26.1
S2 G-I	E15.5 spinal cord	1.302	0.052	20.0	17.8	22.6
S2 J,K	Cortex EGFP	1.206	0.075	16.1	13.5	19.1
S2 J,K	Spinal cord EGFP	1.393	0.013	24.7	24.0	25.5

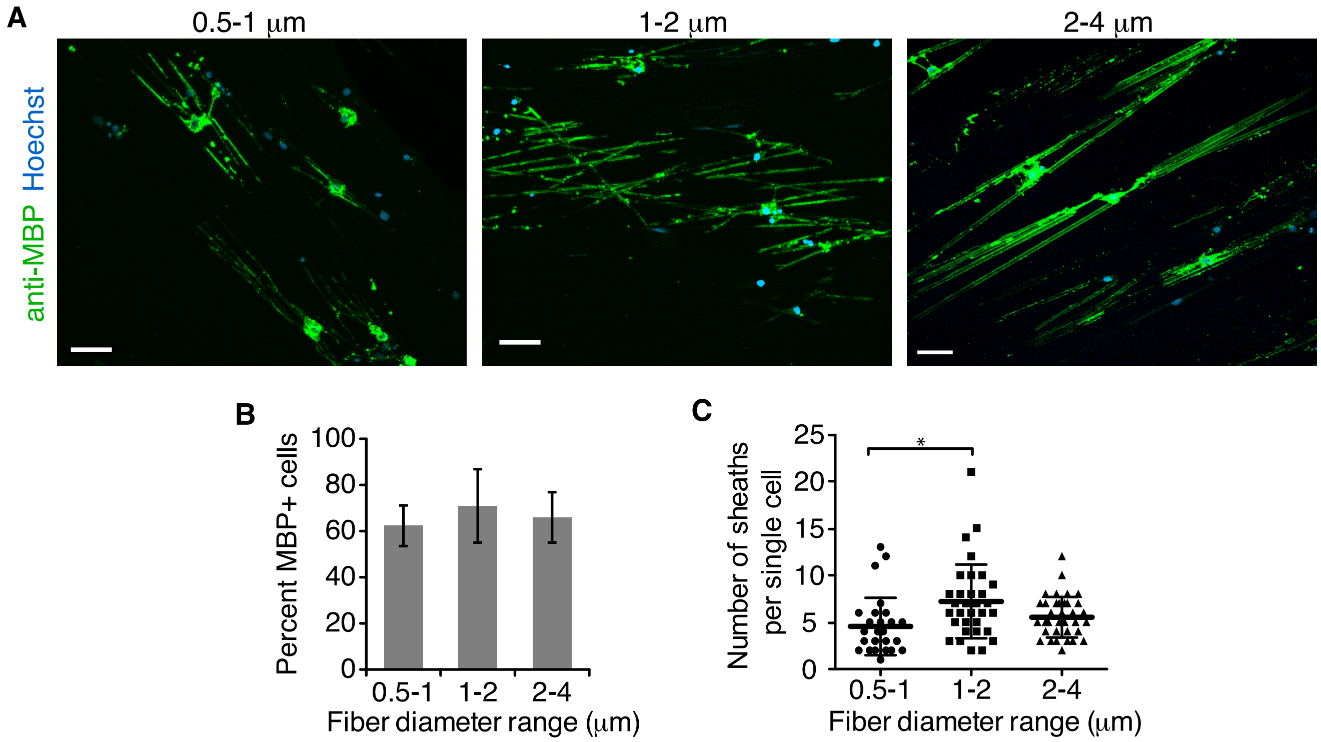

A
0.5-1 μm
1-2 μm
2-4 μm
anti-MBP Hoechst
B
Percent MBP+ cells
100
80
60
40
20
0
0.5-1
1-2
2-4
Fiber diameter range (μm)
C
Number of sheaths per single cell
25
20
15
10
5
0
*
0.5-1
1-2
2-4
Fiber diameter range (μm)

Figure S3. Oligodendrocytes have the ability to sense diameters to generate longer sheath lengths. Also see Figure 3. **(A)** Confocal stacks of oligodendrocytes cultured 14 days on varied diameter microfibers, as indicated. Scale bars, 40 μm. **(B)** The fraction of differentiated cells (MBP+) is not affected by microfiber diameter at Day 14. A minimum of 100 cells was counted for each diameter. Mean with standard deviation is shown from three experiments, each with pooled cells from >3 animals. **(C)** Sheath number formed by individual oligodendrocytes is shown with average and standard deviation indicated. At least 28 single cells were analyzed for each diameter from three experiments, each with pooled cells from >3 animals. Sheath number formed is slightly reduced on fiber diameters of less than one micron (* $p < 0.01$ Kruskal-Wallis, Dunn's multiple comparison test), but not significantly different on fiber diameters greater than one micron.

Table S3. Average sheath lengths with standard deviation.

The average log length and standard deviation were used for statistical tests. The equivalent mean sheath length with lower and upper sheath lengths (mean + or – one standard deviation) is shown, calculated from the mean log(length) ± standard deviation of log(length).

Related figure	Condition	Mean log length	Standard deviation log length	Mean length (microns)	Lower length (microns)	Upper length (microns)
3 A,C	Cortex, 0.5-1 μm diameter	0.893	0.118	7.8	6.0	10.2
3 A,C	Cortex, 1-2 μm diameter	1.091	0.150	12.3	8.7	17.4
3 A,C	Cortex, 2-4 μm diameter	1.447	0.093	28.0	22.6	34.7
3 B,C	Spinal cord, 0.5-1 μm diameter	1.099	0.074	12.6	10.6	14.9
3 B,C	Spinal cord, 1-2 μm diameter	1.353	0.061	22.5	19.6	25.9
3 B,C	Spinal cord, 2-4 μm diameter	1.744	0.054	55.5	49.0	62.7

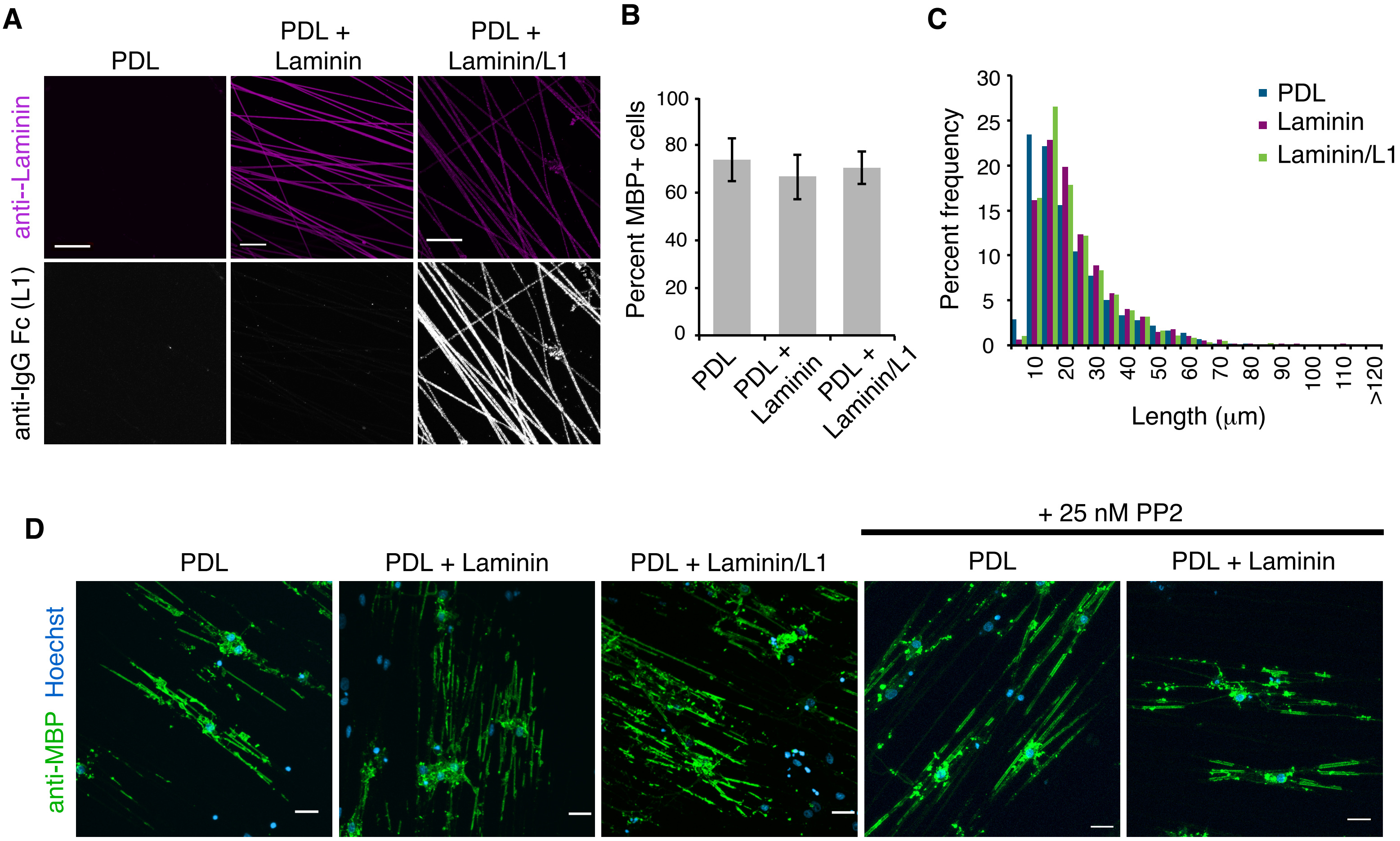
A
PDL
PDL + Laminin
PDL + Laminin/L1
anti--Laminin
anti-IgG Fc (L1)
B
Percent MBP+ cells
100
80
60
40
20
0
PDL
PDL + Laminin
PDL + Laminin/L1
C
Percent frequency
30
25
20
15
10
5
0
PDL
Laminin
Laminin/L1
10
20
30
40
50
60
70
80
90
100
110
>120
Length (μm)
D
+ 25 nM PP2
PDL
PDL + Laminin
PDL + Laminin/L1
PDL
PDL + Laminin
anti-MBP Hoechst

Figure S4. The number of sheaths produced by cortical oligodendrocytes but not sheath lengths is increased on laminin-coated microfibers. Also see Figure 4. **(A)** Confocal images verifying microfiber coating with laminin (anti-laminin) and L1 (anti-IgG Fc) recombinant proteins. Scale bars, 40 μm. (**B)** The percent of differentiated (MBP+) cortical oligodendrocytes is not changed in the presence of laminin or laminin+L1 coating. **(C)** Histogram of cortical oligodendrocyte sheath lengths. More than 900 sheaths were measured per condition from 3 experiments, each with pooled cells from >3 animals added. **(D)** Confocal images of cortical oligodendrocytes cultured 14 days on 1-2 μm microfibers coated as indicated ± addition of the Fyn inhibitor PP2. Scale bars, 20 μm.

Table S4. Average sheath lengths with standard deviation.

The mean log length and standard deviation were used for statistical tests. The equivalent mean sheath length with lower and upper sheath lengths (mean + or – one standard deviation) is shown, calculated from the mean log(length) ± standard deviation of log(length).

Related figure	Condition	Mean log length	Standard deviation log length	Mean length (microns)	Lower length (microns)	Upper length (microns)
4 B, S4 C	PDL	1.196	0.077	15.7	13.2	18.7
4 B, S4 C	PDL + Laminin	1.196	0.097	15.7	12.6	19.6
4 B, S4 C	PDL + Laminin/L1	1.223	0.021	16.7	15.9	17.5

Supplemental Experimental Procedures

Primary Cell Culture

All primary cell isolation was done in accordance with regulations of the Animal (Scientific Procedures) Act under an issued UK Home Office project license. Oligodendrocyte precursor cells were isolated from pooled P0-P2 Sprague-Dawley neonatal rat cerebral cortices as well as P0-P2 or E15.5 spinal cords from one or more litters. Cortices and spinal cords were isolated, meninges removed, minced, and cells were dissociated for 60 min at 37°C with 1.2 U/mL papain (Worthington), 0.1 mg/mL L-cysteine (Sigma-Aldrich) and 0.40 mg/mL DNase I (Sigma-Aldrich). The resulting mixed glial cultures were plated onto poly-D-lysine (PDL) coated flasks and cultured at 37°C in 7.5% CO_2 in DMEM (Invitrogen 41966029), 10% fetal bovine serum (FBS, Invitrogen) and 1% penicillin/streptomycin (pen/strep, Invitrogen). Mixed glial cultures were separated after 10 days by mechanical shaking at 240 rpm for 1 h to remove microglia followed by additional 18 h shaking [S1]. Cells collected were plated onto petri dishes 20 min for differential adhesion to further remove microglia and astrocytes. The enriched oligodendrocyte precursor cells (OPCs) were collected and plated at 35,000 onto fibers or 70,000 onto DRG neurons in myelin media: 50:50 DMEM:Neurobasal Media, B27 (Invitrogen), 5 μg/mL N-acetyl cysteine, and 10 ng/mL D-biotin, ITS , and modified Sato (100 μg/mL BSA fraction V, 60 ng/ml Progesterone, 16 μg/ml Putrecsine, 400 ng/mL Tri-iodothyroxine, 400 ng/mL L-Thyroxine; reagents from Sigma-Aldrich).

Dorsal root ganglia neurons (DRGs) were isolated and cultured as previously described [S2]. DRGs were removed from a litter of E15.5 Sprague Dawley rat embryos and dissociated with 1.2 U/mL papain, 0.24 mg/mL L-cysteine and 0.40 mg/mL DNase I for 60 min at 37°C. Cells were

plated at 150,000 cells per 18 mm coverslip, coated with PDL and growth factor-reduced Matrigel (BD Biosciences). DRG neurons were cultured at 37°C, 7.5% CO_2 in DMEM with 10% FBS, 1% pen/strep, and 100 ng/mL nerve growth factor (NGF, AbD Serotec). To reduce contaminating cell growth, cultures were pulse-treated 3 times with 10 μM 5-fluoro-2'-deoxyuridine (Sigma-Aldrich) for 3 days each on/off, starting within 16 h after plating. After 21 days, the media was changed to the appropriate myelination media.

Primary rat Schwann cells were obtained from pooled P0-P2 neonatal Sprague-Dawley rat sciatic nerves. Sciatic nerves were dissociated with 1 mg/mL Collagenase II (Invitrogen 17101-015) for 30 min at 37°C, followed by Collagenase II/Trypsin dissociation at 37°C for 30 min. Cells were immunopanned twice using an OX7 supernatant (provided generously by the Brophy lab, The University of Edinburgh, UK)-coated plate before culturing on a PDL-coated plate at 37°C 7.5% CO_2 in DMEM, 10% FBS, 1% pen/strep, 10 ng/mL Neuregulin1 (Nrg1, R&D Systems 396-HB), and 2 μg/mL forskolin (Sigma-Aldrich F6886). Schwann cells were maintained for a maximum of three passages after plating, and used at 35,000 or 70,000 cells per fiber or DRG co-culture, respectively. For myelination, Schwann cells were cultured in DMEM/F-12 (Invitrogen 11320033) supplemented with Sato, ITS, pen/strep, 2% FBS, and 50 μg/mL freshly prepared ascorbic acid.

For labelled cell experiments, 250,000 oligodendrocyte precursor cells were plated into each well of a 6-well plate with DMEM, ITS, modified Sato's medium with 0.5% FBS, 10 ng/ml PDGF, and 10 ng/ml FGF for a day. After 24 h cells were incubated overnight with lentivirus at 10 MOI for EGFP expression. Lentiviruses were obtained from the Biomolecular Core Facility in the

MRC Human Reproductive Sciences Unit (The University of Edinburgh, UK). Cells were collected by TrypLe (Invitrogen) detachment and counted. EGFP labelled cells were mixed equally with unlabeled cells (i.e. EGFP expressing cortical OPCs with unlabeled spinal cord OPCs, and vice versa). Cells were then cultured on fibers as above.

Slice Cultures

The cerebellum of a P3 *shiverer* mouse (*Mbp*^{shi}/*Mbp*^{shi} from the C3Fe.SWV-*Mbp*^{shi}/J genetic background) was sectioned sagitally in 300 μm slices on a McIlwain tissue chopper, and plated onto Millipore-Millicel-CM mesh inserts in 6-well culture plates. Media was composed of 50% minimal essential media, 25% heat-inactivated horse serum, 25% Earle's balanced salt solution, 6.5 mg/ml glucose (Sigma), 1% pen/strep, and Glutamax (Invitrogen), and was changed every 2-3 days. After 2 days in culture, 50,000 EGFP-expressing cortical or spinal cord oligodendrocytes were added. Fourteen day after addition of OPCs, slices were fixed 1 h in 4% PFA in PBS, washed 3 x 1 h in PBS, blocked for 1 h. Primary antibodies were incubated for 36 h at 4°C, slices were washed, and fluorescently conjugated antibody was applied overnight at 4°C. Slices were mounted onto glass slides using Fluoromount-G (Southern Biotech).

Electrospun Fibers

Custom parallel-aligned electrospun fibers composed of poly-L-lactic acid were synthesized and suspended by fitting into 12-well plate inserts by The Electrospinning Company. Fiber diameter ranges that are known to promote myelin sheath formation in vivo were used [S3], at densities that produced < 10 μm spacing. Fiber diameter ranges of 0.5-1 μm, 1-2 μm, and 2-4 μm were

generated and were verified with scanning electron microscopy (SEM, Figure S1A). Prior to use, inserts were soaked in 70% ethanol, washed, coated 1 h at 37°C with 5 μg/mL PDL, followed by three washes to remove excess PDL. For laminin experiments, fibers were further coated by a solution of 10 μg/mL human placental laminin (Sigma, L6274) with or without 5 μg/mL L1 (R&D Systems, 777-NC) overnight at room temperature, followed by three washes.

Immunofluorescence

Cells were washed once with phosphate buffered saline, pH 7.4 (PBS), fixed in 4% paraformaldehyde/PBS, followed by PBS washes, and permeabilization in 0.1% TritonX-100 in PBS. Primary antibodies were diluted in PBS, as indicated below, and incubated overnight at 4°C. Primary antibodies used were: rat anti-myelin basic protein (MBP) 1:250 (AbD Serotec), chicken anti-neurofilament (NfH) at 1:1000 (Sigma-Aldrich), rabbit or mouse anti-NG2 1:200 or 1:100 (Millipore), mouse anti-MOG 1:100 (Millipore), rabbit anti-S100 1:250 (Dako), rabbit anti-GFAP 1:500 (Dako), goat anti-human IgF Fc 1:250 (Jackson Immunoresearch), chicken anti-GFP 1:500 (Abcam), and mouse anti-laminin 1:100 (Millipore). Cells were washed in PBS again and incubated 1 h with Alexafluor 488, 568, or 647 conjugated secondary antibodies against rat, rabbit, mouse, goat, or chicken (Invitrogen), used at a 1:1000 dilution. After PBS washes, cells were stained with 5 μg/mL Hoechst (Sigma-Aldrich), washed again and mounted with Fluoromount G (Southern Biotech) with cover glass onto glass slides.

Fluorescence Imaging and Image Analysis

Confocal images were obtained on a Leica SPE confocal or SP8 confocal scanning microscope, with 20x/NA0.5, 40x oil/NA1.3, and 40x oil/NA1.25 objectives. Confocal stacks of 0.38-0.5 μm z-steps were taken as necessary at 1024x1024. At least 10 random areas across each coverslip were imaged. The same settings (gain, binning, z-steps, etc.) were used between coverslips in a single experiment. Any brightness or contrast adjustments were done uniformly across all images from a single experiment.

The number of MBP-positive cells, in combination with Hoechst nuclei staining, was counted with at least 80 cells counted per coverslip per experiment. Similarly, NG2 or GFAP positive cells were counted. The percent of wrapping MBP+ cells was determined by counting the number of MBP+ cells that were forming at least one sheath surrounding fibers. For sheath measurements, a sheath was defined as a continuous, smooth tube of MBP staining surrounding an axon or fiber (Figure 1B). For all experiments, except those comparing fiber diameters, lengths were measured from blinded confocal images by drawing and acquiring length measurements in Fiji [S4] version of ImageJ (NIH). For initial experiments comparing spinal cord and cortical oligodendrocytes, experiments were reproduced by two individuals. For experiments comparing fiber diameters, however, it was not possible to blind experiments, as the diameters were evident upon viewing the images. Independent experiments (i.e. cells pooled from different rat litters on different days) were conducted with a minimum of 200 sheaths measured for microfiber experiments (as indicated in figure legends). Statistical analysis was done in GraphPad Prism 5. For differentiation analysis, one-way ANOVAs were conducted. For comparison of multiple length distributions, the Gaussian distribution of log lengths was

used to compare mean log lengths between experiments by one-way ANOVA with Tukey's multiple comparison post-testing. For comparison of two length distributions (spinal cord versus cortical oligodendrocytes), a paired two-tailed t-test was used for mean log lengths. For single cell analysis (sheath number per single cell), the number of sheaths for each individual MBP-positive cell was measured and counted. A normal distribution was not assumed for sheath number; therefore a Kruskall-Wallis test with Dunn's multiple comparison post-testing was used.

Electron Microscopy

Cells were briefly washed in 0.1 M phosphate buffer, pH 7.2 (PB), followed by 2 h fixation in 2.5% glutaraldehyde/4% paraformaldehyde in PB. Cells were washed in PB then fixed 1 h at $4^{o}C$ with 1% Osmium tetroxide (Electron Microscopy Sciences) in PB. Cells were rinsed further in PB, and then dehydrated with a gradient of 50, 75, 90, and 100% ethanol 2 times each. For transmission electron microscopy, after dehydration, samples were placed into 50:50 mix of resin:ethanol for an hour, followed by multiple resin changes over 48 h. Resin was either Agar100 or LR White (Agar Scientific). Resin was then changed and cured for a minimum of 24 h at $60^{o}C$. Ultrathin 60 nm sections were cut, followed by Uranyl Acetate and Lead Citrate post-staining (Electron Microscopy lab, Institute of Molecular Plant Sciences, The University of Edinburgh, UK). Images were acquired on a Phillips CM120 transmission electron microscope equipped with a Gatan Orius CCD camera. For scanning electron microscopy, samples were critical point dried post-ethanol dehydration, and mounted on aluminum stubs for palladium/gold sputter coating. SEM images were acquired on a XL30CP scanning electron microscope (Philips). The number of contacting, wrapping (single layer), and multilayer wrapping occurrences was counted from images of 1-2 micron fibers wherever an oligodendrocyte process

was in contact with fibers (contact with cell bodies were excluded). A total of 350 across 7 biological replicates (different cell isolations on different days) were counted to determine percent for each category.

Supplemental References

S1. McCarthy, K. D., and de Vellis, J. (1980). Preparation of separate astroglial and oligodendroglial cell cultures from rat cerebral tissue. J. Cell. Biol. *85*, 890–902.

S2. Laursen, L. S., Chan, C. W., and ffrench-Constant, C. (2009). An integrin-contactin complex regulates CNS myelination by differential Fyn phosphorylation. J. Neurosci. *29*, 9174–9185.

S3. Hildebrand, C., Remahl, S., Persson, H., and Bjartmar, C. (1993). Myelinated nerve fibres in the CNS. Prog. Neurobiol. *40*, 319–384.

S4. Schindelin, J., Arganda-Carreras, I., Frise, E., Kaynig, V., Longair, M., Pietzsch, T., Preibisch, S., Rueden, C., Saalfeld, S., Schmid, B., et al. (2012). Fiji: an open-source platform for biological-image analysis. Nat. Meth. *9*, 676–682.
