# Supplementary material for: CNS Myelin Sheath Lengths Are an Intrinsic Property of Oligodendrocytes
Source: Curr Biol. 2015 Sep 21;25(18):2411–6. doi: 10.1016/j.cub.2015.07.056 (PMC4580335; doi:10.1016/j.cub.2015.07.056)
Supplement: Document S2. Article plus Supplemental Information [file mmc2.pdf]

Current Biology

Report

CNS Myelin Sheath Lengths Are an Intrinsic Property of Oligodendrocytes

Graphical Abstract

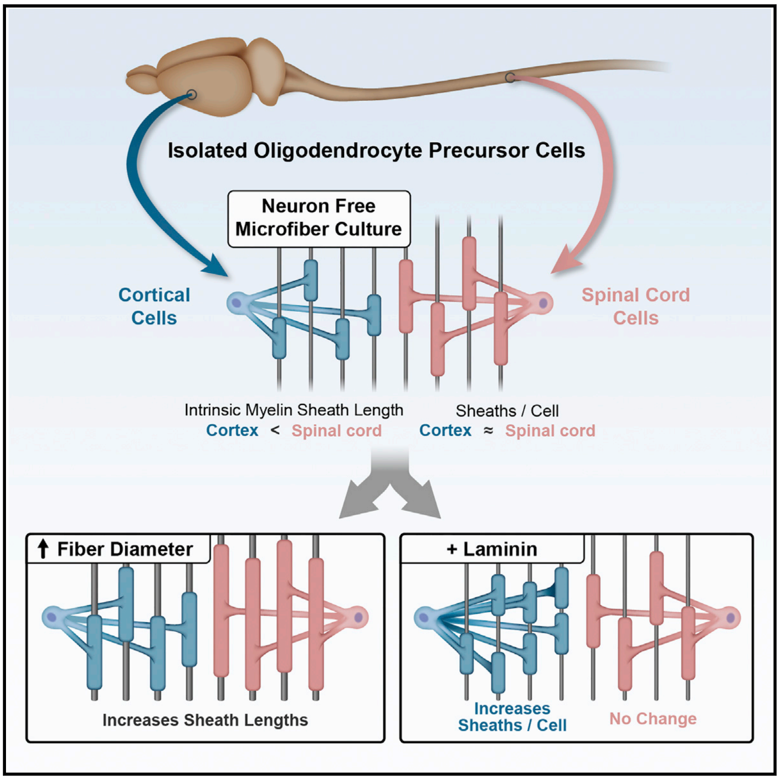

Highlights

- Oligodendrocytes, not Schwann cells, form myelin sheaths without axonal signals
- Oligodendrocytes sense diameter, increasing sheath length with larger fibers
- Oligodendrocytes from cortex and spinal cord form sheaths of different lengths
- Regional differences in sheath lengths may initially be hard-wired

Authors

Marie E. Bechler, Lauren Byrne, Charles ffrench-Constant

Correspondence

mbechler@staffmail.ed.ac.uk

In Brief

Much of the mammalian brain comprises axons wrapped by myelin sheaths, whose length determines conduction velocity. Bechler et al. overturn the long-held view that sheath-forming oligodendrocytes are all the same. Oligodendrocytes from different regions generate sheath lengths on microfibers and neurons that reflect their in vivo origin.

Bechler et al., 2015, Current Biology *25*, 2411–2416
September 21, 2015 ©2015 The Authors
http://dx.doi.org/10.1016/j.cub.2015.07.056

Current Biology
Report

CellPress

CNS Myelin Sheath Lengths Are an Intrinsic Property of Oligodendrocytes

Marie E. Bechler,[1,*] Lauren Byrne,[1] and Charles ffrench-Constant[1]
[1]MRC Centre for Regenerative Medicine, The University of Edinburgh, 5 Little France Drive, Edinburgh EH16 4UU, UK
*Correspondence: mbechler@staffmail.ed.ac.uk
http://dx.doi.org/10.1016/j.cub.2015.07.056
This is an open access article under the CC BY license (http://creativecommons.org/licenses/by/4.0/).

SUMMARY

Since Río-Hortega's description of oligodendrocyte morphologies nearly a century ago, many studies have observed myelin sheath-length diversity between CNS regions [1–3]. Myelin sheath length directly impacts axonal conduction velocity by influencing the spacing between nodes of Ranvier. Such differences likely affect neural signal coordination and synchronization [4]. What accounts for regional differences in myelin sheath lengths is unknown; are myelin sheath lengths determined solely by axons or do intrinsic properties of different oligodendrocyte precursor cell populations affect length? The prevailing view is that axons provide molecular cues necessary for oligodendrocyte myelination and appropriate sheath lengths. This view is based upon the observation that axon diameters correlate with myelin sheath length [1, 5, 6], as well as reports that PNS axonal neuregulin-1 type III regulates the initiation and properties of Schwann cell myelin sheaths [7, 8]. However, in the CNS, no such instructive molecules have been shown to be required, and increasing in vitro evidence supports an oligodendrocyte-driven, neuron-independent ability to differentiate and form initial sheaths [9–12]. We test this alternative signal-independent hypothesis—that variation in internode lengths reflects regional oligodendrocyte-intrinsic properties. Using microfibers, we find that oligodendrocytes have a remarkable ability to self-regulate the formation of compact, multilamellar myelin and generate sheaths of physiological length. Our results show that oligodendrocytes respond to fiber diameters and that spinal cord oligodendrocytes generate longer sheaths than cortical oligodendrocytes on fibers, co-cultures, and explants, revealing that oligodendrocytes have regional identity and generate different sheath lengths that mirror internodes in vivo.

RESULTS

In order to distinguish oligodendrocyte-driven versus axon-instructed myelin sheath formation, we established a neuron-free, three-dimensional culture system with poly-L-lactic acid (PLA) microfibers (Figure S1A). Most cortical oligodendrocyte precursor cells differentiated into oligodendrocytes, as seen by myelin basic protein (MBP) expression and a corresponding reduction in NG2 at 7 days (Figure S1B). These oligodendrocytes formed MBP^+ sheaths surrounding microfibers (Figures 1A and 1B), as in previous reports [12], with 90% ± 4% of MBP^+ cells ensheathing by 14 days. We verified that sheaths formed on fibers contain other late oligodendrocyte markers, such as myelin oligodendrocyte glycoprotein (MOG), by immunolabeling (Figure S1C). We also confirmed that they formed multi-layered compacted membranes characteristic of myelin sheaths by electron microscopy (Figure 1C), with the majority of processes contacting fibers generating sheaths of two to ten layers (Figure S1D). Whereas both Schwann cells and oligodendrocytes myelinated dorsal root ganglia (DRG) neurons (Figure 1A), Schwann cells did not differentiate or wrap the microfibers when cultured under identical conditions (Figure 1A), showing a fundamental difference in their requirements for axonal signals in myelination. Next, we examined whether isolated cortical oligodendrocytes produce myelin sheath lengths equivalent to that found on axons. MBP^+ sheaths were measured from oligodendrocyte cultures with DRG neurons or comparable caliber (1–2 μm) microfibers [13]. Within 7 days, sheath lengths were identical to those seen in co-culture with DRG neurons (Figures 1D and 1E; Table S1) and were comparable to reported in vivo cortex distributions [14].

In vivo, spinal cord internodes are on average twice the length of cortical internodes [1, 15]. To determine whether oligodendrocytes possess regional identity that governs the lengths of sheaths, we examined oligodendrocyte precursor cells from spinal cord and cortex in the absence of environmental variation, by culturing cells under identical conditions on 1–2 μm microfibers. Oligodendrocyte precursor cells isolated from spinal cord showed similar purity, density, differentiation, and ensheathment on microfibers to cortical oligodendrocytes (Figures S2A–S2D). Surprisingly though, the sheath lengths formed by spinal cord oligodendrocytes were significantly longer than cortical oligodendrocytes (Figures 2A, 2B, and S2F; Table S2). By contrast, the number of sheaths formed was not significantly different between the two populations (Figure S2E). This shows for the first time that oligodendrocytes from two CNS regions are not the same but instead have intrinsic differences that dictate relative sheath lengths.

We confirmed that the longer sheaths in the neonatal spinal cord cells did not result from the differences in the timing of oligodendrocyte development in the two tissues (as

CrossMark

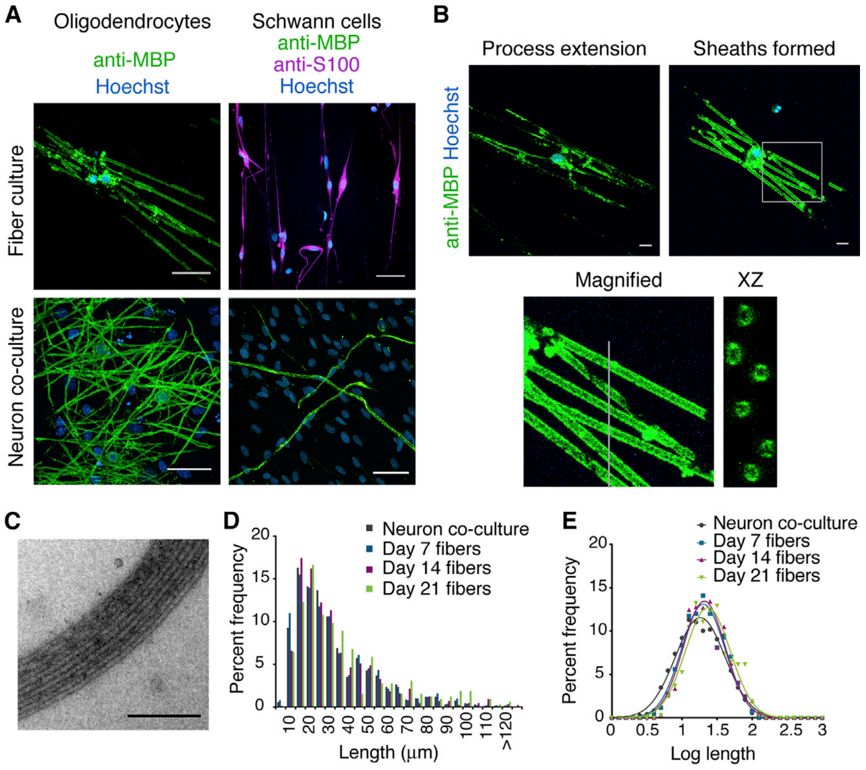

Figure 1. Oligodendrocytes Have the Unique, Intrinsic Capability to Generate Compact Membrane Sheaths and Physiological Internode Lengths on Microfibers

(A) Confocal stacks of rat primary cortical oligodendrocytes or Schwann cells cultured 14 or 21 days, respectively, on 1–2 μm microfibers or neurons. The scale bars represent 40 μm.

(B) Representative confocal images showing the distinction between process extension and sheath formation. Magnified and cross-section (xz) images are shown on the bottom. The scale bars represent 10 μm.

(C) Electron micrographs of multi-layered oligodendrocyte membranes around microfibers by 14 days. The scale bar represents 200 nm.

(D) Sheath length histogram showing percent frequency in 5 μm bins. More than 700 sheaths were measured from three experiments, each with pooled cells from greater than six animals.

(E) Log transformation of lengths shows Gaussian distributions with no significant difference in mean (one-way ANOVA).

See also Figure S1 and Table S1.

myelination starts earlier in the spinal cord than in the cortex) by repeating the analysis using spinal cord oligodendrocyte precursors at E15.5 prior to the onset of myelination in this region. Once again, these cells formed longer sheaths (Figures S2G–S2I). We also confirmed the oligodendrocyte-intrinsic nature of sheath lengths in three ways. First, we mixed cortical and spinal cord oligodendrocyte precursor cells in a single culture, using lentiviral expression of EGFP to distinguish the two populations (Figures S2J and S2K). Second, we cultured the cortical and spinal cord oligodendrocyte precursor cells on DRG neurons (Figures 2C and 2D). Third, we added the two populations to cerebellar slices of *shiverer* mutant mice that lack MBP, enabling the MBP^{+} myelin sheaths formed by the added cells to be visualized and measured within CNS tissue (Figures 2E, 2F, and S2L; Table S2). In all cases, the spinal cord cells generated longer sheaths. Together, these show that external signals from other CNS cell types do not contribute to the signaling responsible for the relative differences in sheath length.

As oligodendrocytes generally form longer internodes on larger-diameter axons in vivo and axon calibers are larger in spinal cord [1], we asked whether oligodendrocytes recognize different caliber axons to generate appropriate-length myelin sheaths in the absence of axonal molecules. To assess the ability of oligodendrocyte processes to respond to purely physical cues, we cultured oligodendrocytes on microfibers of varied diameter ranges: 0.5–1 μm; 1–2 μm; and 2–4 μm (Figures S1A and S3A). The percent of cells expressing MBP was not affected (Figure S3B). The number of sheaths formed was slightly reduced on the smallest-caliber microfibers but did not differ significantly on fibers greater than one micron in diameter (Figure S3C). Cortical oligodendrocytes cultured 14 days on microfibers demonstrated a significant increase in sheath lengths on larger-diameter microfibers (Figures 3A and S3A; Table S3), showing a remarkable ability for oligodendrocytes to respond to caliber by changing sheath lengths. Similarly, spinal cord oligodendrocytes had longer sheath lengths on larger microfiber diameters (Figure 3B; Table S3). Importantly, longer spinal cord sheath lengths, relative to cortex, were found on all microfiber diameters (Figures 3A–3C), demonstrating that, in addition to any effect of the larger-diameter axons in spinal cord, intrinsic differences in the control of cell shape between oligodendrocyte populations result in internode length differences.

Several axonal signals, such as laminin2, have been shown to modulate myelin sheath formation [16]. We asked whether axonal molecules known to enhance myelination modulate the oligodendrocyte-intrinsic program controlling myelin sheath lengths and whether there are region-specific responses. Axonal laminin2 and neuronal cell adhesion molecule L1 have been shown to enhance the formation of myelin sheaths in cortical oligodendrocytes through activation of the Src family kinase Fyn [16]. Therefore, we examined whether PLA microfibers coated with laminin with and without L1 affect sheath formation. One to two micron microfibers were coated with poly-D-lysine (PDL) with or without laminin and L1 (Figure S4A). No significant effect on differentiation was found (cells expressing MBP; Figure S4B). Cortical oligodendrocyte sheath lengths were also unaffected by the addition of laminin or laminin in combination with L1 (Figures 4B, S4C, and S4D; Table S4). However, laminin coating increased the number of sheaths per cortical oligodendrocyte, which was not seen in the presence of the Fyn inhibitor PP2 (Figure 4A). This increase in sheath number, but not length, is consistent with our previous results with activated Fyn expression in zebrafish

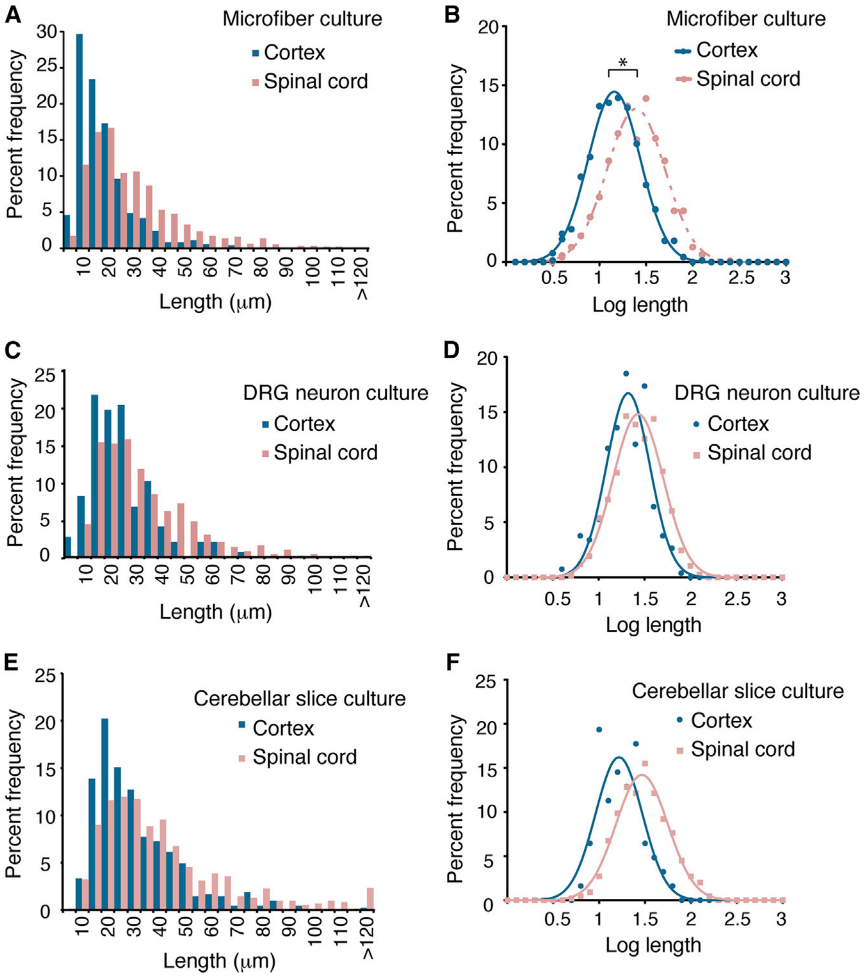

Figure 2. Oligodendrocytes Have Regional Identity that Determines Relative Internode Lengths

(A) Histogram of sheath lengths formed by spinal cord- or cortex-isolated oligodendrocytes cultured 14 days on 1–2 μm microfibers.

(B) Log sheath lengths on 1–2 μm fibers. * indicates $p < 0.01$; two-tailed t test of mean log lengths. More than 700 sheaths were measured from five experiments, each with pooled cells from greater than three animals.

(C) Histogram of sheath lengths formed by either spinal cord- or cortex-isolated oligodendrocytes cultured 14 days on DRG neurons. For each region, more than 250 sheaths were measured from two DRG cultures with a pool of cells from greater than five animals.

(D) Log sheath length plot for cultures on DRG neurons.

(E) Histogram of sheath lengths formed by either EGFP-expressing spinal cord or cortical oligodendrocytes added to *shiverer* mouse cerebellar slice cultures for 14 days. For each region, more than 400 sheaths were measured from two slices with a pool of cells from greater than five animals.

(F) Log sheath length plot for oligodendrocytes cultured on *shiverer* cerebellar slices.

See also Figure S2 and Table S2.

oligodendrocytes [17]. To address whether oligodendrocytes from the cortex and spinal cord respond similarly to laminin, we cultured spinal cord or cortical oligodendrocytes on PDL- or PDL and laminin-coated microfibers. Unlike cortical oligodendrocytes, the number of sheaths formed by individual spinal cord oligodendrocytes was not increased with laminin-coated microfibers (Figure 4C). These results further highlight regional differences between cortical and spinal cord oligodendrocytes, demonstrating divergent responses to molecules that modulate myelin sheath formation.

DISCUSSION

Using a microfiber culture system in which oligodendrocytes ensheath inert fibers [12], we find that these myelinating cells of the CNS have a remarkable intrinsic capability to form compact sheaths of expected lengths without molecular instruction from axons. We further find that oligodendrocyte precursor cells are programmed to generate sheath lengths that reflect their in vivo origin, demonstrating that oligodendrocyte precursor cells have acquired a regional identity prior to differentiation and that this identity instructs the signaling pathways that determine sheath length. Together, these intrinsic properties would, without any signals from axonally derived molecules, generate a diversity of oligodendrocyte morphologies within the CNS that has been documented for nearly a century without any understanding of mechanism.

What then is the role of the axon in regulating myelination? We suggest that, contrary to the long-held belief that axonal signals actively direct (and are required for) the process of myelin sheath formation, they are rather responsible for modifying a "hard-wired" pattern established by the intrinsic properties above. Our work shows two mechanisms by which this adaption might be achieved: first by axon size, with oligodendrocytes capable of responding to increased fiber caliber by increasing sheath length, and second by axonal molecules that adapt thickness or sheath number. Myelin thickness has been shown to change with altered levels of neuregulin-1 type III [18, 19]. The number of sheaths formed by oligodendrocytes can be increased by activating Fyn signaling [17], and here, we find that laminin-activated Fyn signaling increases sheath number, but not lengths, in cortical oligodendrocytes. The observation of greater variance of sheath lengths in the cerebellar slice experiments (shown in Figure 2E) points to a role for further signals from an in vivo milieu. The signaling mechanisms by which the oligodendrocytes themselves regulate sheath length remain unknown, and the link between these pathways, a mechanical signal sensitive to axon diameter as illustrated by our results, and other in vivo signals will be important points for further work.

Importantly, these signaling pathways that affect sheath properties may differ in regions of the CNS. Not only do

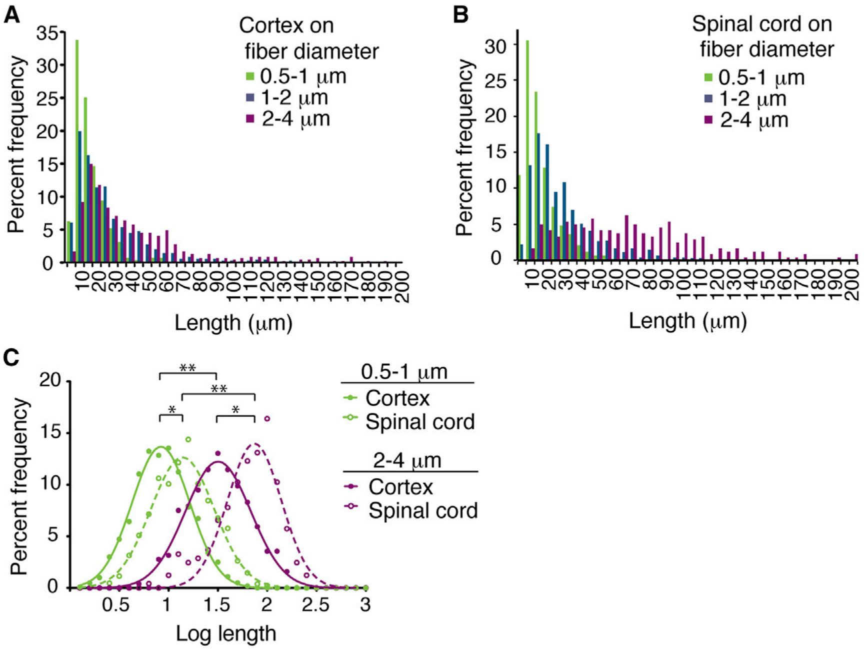

Figure 3. Regional Identity and Physical Cues Explain Differences in Internode Lengths between Cortex and Spinal Cord

(A) Sheath lengths of cortical oligodendrocytes increase with increased diameter microfibers.

(B) Spinal cord oligodendrocyte sheath lengths also increase with increased diameter microfibers.

(C) Log sheath lengths show significant differences between spinal cord and cortical oligodendrocytes on all diameter ranges of microfibers (* indicates $p < 0.03$; two-tailed t test for mean log lengths) and across microfiber diameters for cells from the same region (** indicates $p < 0.01$; one-way ANOVA for mean log lengths). More than 250 sheaths were measured from four experiments, each with pooled cells from greater than three animals.

See also Figure S3 and Table S3.

developmentally different sources of oligodendrocyte precursors [20, 21] show regional identity reflected by sheath lengths, we find the response to the environmental cue laminin is also region specific. Our result that cortical, but not spinal cord, oligodendrocytes increase sheath number in response to laminin leads to the conclusion that integrin-activated Fyn signaling is not instructive for myelination in spinal cord oligodendrocytes. This conclusion is consistent with the observations that spinal cord myelination is normal in Fyn KO mice whereas myelin in the forebrain is diminished and that mice lacking laminin $\alpha 2$ have a decrease in myelinated axons in the brain but show very little effect for spinal cord myelination [22, 23].

An intrinsic pathway that sets up a pre-determined pattern of myelination followed by extrinsic cues that then modify the myelin sheaths points to a flexible developmental mechanism. Such a mechanism would establish an initial network capable of rapid neuronal conduction that can then be sculpted by environmental signals. Our demonstration that sheath length and number are regulated independently (as evidenced by the effects of axon size on sheath length and laminin on sheath number), together with the work of others showing that neuronal activity can affect myelin thickness [24–26], emphasizes the potential adaptability of the cortical oligodendrocytes in response to these environmental signals. Together with the demonstration of an intrinsic program to establish a basic pattern of myelination, it suggests a sequence analogous to synaptogenesis in the CNS, in which a hard-wired pattern established during development is adapted by experience-dependent activity. Each of the adaptable variables (length, number, and thickness) will regulate conduction velocities along single axons, and it will be important in future work to establish, by a combination of experimental approaches and computational modeling, the extent to which adaptive myelination occurs and could contribute to the plasticity of the CNS that underpins higher cognitive functions such as learning.

SUPPLEMENTAL INFORMATION

Supplemental Information includes Supplemental Experimental Procedures, four figures, and four tables and can be found with this article online at http://dx.doi.org/10.1016/j.cub.2015.07.056.

ACKNOWLEDGMENTS

We thank M. Grove (Brophy laboratory) and S. Hess (Rambukkana laboratory) for reagents and assistance with Schwann cell cultures. We thank V. Miron, M. Harrisingh, M. Swire, A. Jarjour, S. Sekizar, N. Vasistha, A. Boyd, R. Lewis, and S. Mitchell for technical assistance. We also thank D. Lyons for comments on the manuscript. This work was supported by the Wellcome Trust and the MRC UK Regenerative Medicine Platform (to C.ff.-C.).

Received: March 26, 2015
Revised: June 24, 2015
Accepted: July 22, 2015
Published: August 27, 2015

REFERENCES

1. Hildebrand, C., Remahl, S., Persson, H., and Bjartmar, C. (1993). Myelinated nerve fibres in the CNS. Prog. Neurobiol. *40*, 319–384.
2. Del Rio-Hortega, P. (1921). Estudios sobre la neuroglia. La glia de escasas radiaciones oligodendroglia. Bol. Real Soc. Esp. Hist. Nat. *21*, 63–92.
3. Del Río-Hortega, P. (1928). Tercera aportación al conocimiento morfológico e interpretación funcional de la oligodendroglía. Mem. Real Soc. Esp. Hist. Nat. *14*, 40–122.
4. Pajevic, S., Basser, P.J., and Fields, R.D. (2014). Role of myelin plasticity in oscillations and synchrony of neuronal activity. Neuroscience *276*, 135–147.
5. Murray, J.A., and Blakemore, W.F. (1980). The relationship between internodal length and fibre diameter in the spinal cord of the cat. J. Neurol. Sci. *45*, 29–41.

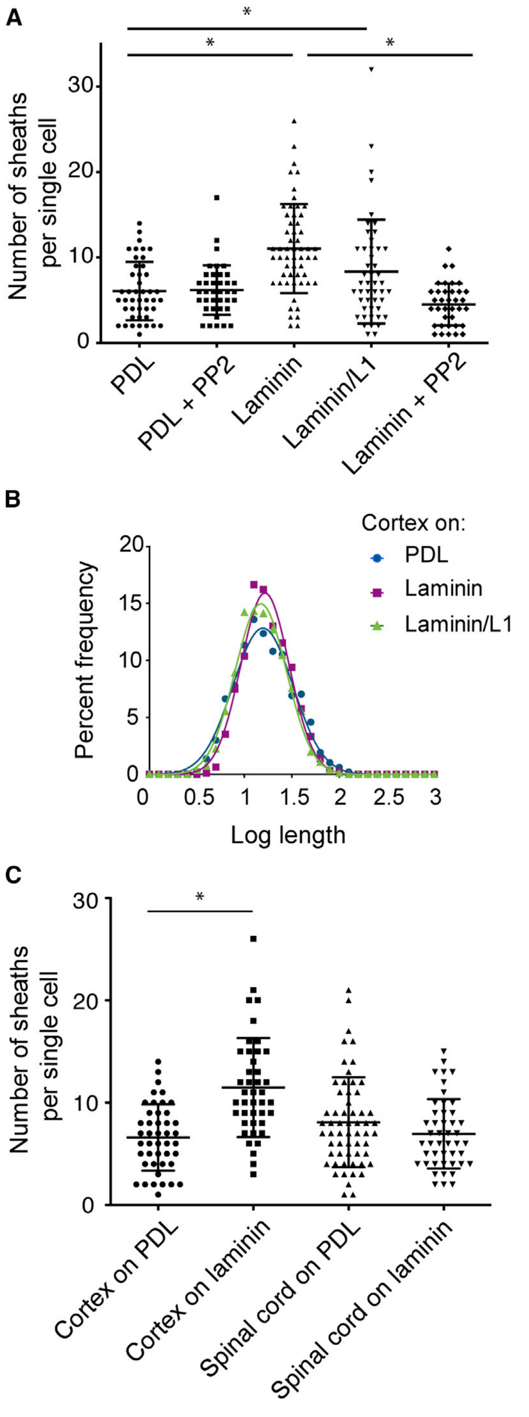

Figure 4. Cortical, but Not Spinal Cord, Oligodendrocytes Respond to Laminin by Producing More Sheaths per Cell

(A) Laminin, dependent on Fyn activity, increases the mean sheaths per single cortical oligodendrocyte 2-fold. Cortical oligodendrocytes were cultured 14 days on poly-D-lysine (PDL)-coated 1–2 μm microfibers with or without laminin or laminin + L1 coating and addition of the Fyn inhibitor PP2. Sheath number was analyzed for individual cells. Average with SD is indicated by bars. * indicates $p < 0.01$, Kruskal-Wallis with Dunn's post-test, with a minimum 42 single cells analyzed for each condition from three experiments, each with pooled cells from greater than three animals.

(B) Log transformation of cortical oligodendrocyte sheath length shows no significant difference in the mean (one-way ANOVA) in the presence of laminin.

(C) The number of sheaths per individual spinal cord oligodendrocyte does not increase in the presence of laminin. At least 42 individual cells were analyzed from three experiments, each with pooled cells from greater than three animals.

See also Figure S4 and Table S4.

6. Ibrahim, M., Butt, A.M., and Berry, M. (1995). Relationship between myelin sheath diameter and internodal length in axons of the anterior medullary velum of the adult rat. J. Neurol. Sci. *133*, 119–127.

7. Michailov, G.V., Sereda, M.W., Brinkmann, B.G., Fischer, T.M., Haug, B., Birchmeier, C., Role, L., Lai, C., Schwab, M.H., and Nave, K.-A. (2004). Axonal neuregulin-1 regulates myelin sheath thickness. Science *304*, 700–703.

8. Taveggia, C., Zanazzi, G., Petrylak, A., Yano, H., Rosenbluth, J., Einheber, S., Xu, X., Esper, R.M., Loeb, J.A., Shrager, P., et al. (2005). Neuregulin-1 type III determines the ensheathment fate of axons. Neuron *47*, 681–694.

9. Mirsky, R., Winter, J., Abney, E.R., Pruss, R.M., Gavrilovic, J., and Raff, M.C. (1980). Myelin-specific proteins and glycolipids in rat Schwann cells and oligodendrocytes in culture. J. Cell Biol. *84*, 483–494.

10. Dubois-Dalcq, M., Behar, T., Hudson, L., and Lazzarini, R.A. (1986). Emergence of three myelin proteins in oligodendrocytes cultured without neurons. J. Cell Biol. *102*, 384–392.

11. Bullock, P.N., and Rome, L.H. (1990). Glass micro-fibers: a model system for study of early events in myelination. J. Neurosci. Res. *27*, 383–393.

12. Lee, S., Leach, M.K., Redmond, S.A., Chong, S.Y.C., Mellon, S.H., Tuck, S.J., Feng, Z.-Q., Corey, J.M., and Chan, J.R. (2012). A culture system to study oligodendrocyte myelination processes using engineered nanofibers. Nat. Methods *9*, 917–922.

13. Windebank, A.J., Wood, P., Bunge, R.P., and Dyck, P.J. (1985). Myelination determines the caliber of dorsal root ganglion neurons in culture. J. Neurosci. *5*, 1563–1569.

14. Murtie, J.C., Macklin, W.B., and Corfas, G. (2007). Morphometric analysis of oligodendrocytes in the adult mouse frontal cortex. J. Neurosci. Res. *85*, 2080–2086.

15. Chong, S.Y.C., Rosenberg, S.S., Fancy, S.P.J., Zhao, C., Shen, Y.-A.A., Hahn, A.T., McGee, A.W., Xu, X., Zheng, B., Zhang, L.I., et al. (2012). Neurite outgrowth inhibitor Nogo-A establishes spatial segregation and extent of oligodendrocyte myelination. Proc. Natl. Acad. Sci. USA *109*, 1299–1304.

16. Laursen, L.S., Chan, C.W., and ffrench-Constant, C. (2009). An integrin-contactin complex regulates CNS myelination by differential Fyn phosphorylation. J. Neurosci. *29*, 9174–9185.

17. Czopka, T., ffrench-Constant, C., and Lyons, D.A. (2013). Individual oligodendrocytes have only a few hours in which to generate new myelin sheaths in vivo. Dev. Cell *25*, 599–609.

18. Taveggia, C., Thaker, P., Petrylak, A., Caporaso, G.L., Toews, A., Falls, D.L., Einheber, S., and Salzer, J.L. (2008). Type III neuregulin-1 promotes oligodendrocyte myelination. Glia *56*, 284–293.

19. Brinkmann, B.G., Agarwal, A., Sereda, M.W., Garratt, A.N., Müller, T., Wende, H., Stassart, R.M., Nawaz, S., Humml, C., Velanac, V., et al. (2008). Neuregulin-1/ErbB signaling serves distinct functions in myelination of the peripheral and central nervous system. Neuron *59*, 581–595.

20. Rowitch, D.H., and Kriegstein, A.R. (2010). Developmental genetics of vertebrate glial-cell specification. Nature *468*, 214–222.

21. Richardson, W.D., Kessaris, N., and Pringle, N. (2006). Oligodendrocyte wars. Nat. Rev. Neurosci. *7*, 11–18.

22. Chun, S.J., Rasband, M.N., Sidman, R.L., Habib, A.A., and Vartanian, T. (2003). Integrin-linked kinase is required for laminin-2-induced oligodendrocyte cell spreading and CNS myelination. J. Cell Biol. *163*, 397–408.

23. Relucio, J., Tzvetanova, I.D., Ao, W., Lindquist, S., and Colognato, H. (2009). Laminin alters fyn regulatory mechanisms and promotes oligodendrocyte development. J. Neurosci. *29*, 11794–11806.

24. Makinodan, M., Rosen, K.M., Ito, S., and Corfas, G. (2012). A critical period for social experience-dependent oligodendrocyte maturation and myelination. Science *337*, 1357–1360.

25. Liu, J., Dietz, K., DeLoyht, J.M., Pedre, X., Kelkar, D., Kaur, J., Vialou, V., Lobo, M.K., Dietz, D.M., Nestler, E.J., et al. (2012). Impaired adult myelination in the prefrontal cortex of socially isolated mice. Nat. Neurosci. *15*, 1621–1623.

26. Gibson, E.M., Purger, D., Mount, C.W., Goldstein, A.K., Lin, G.L., Wood, L.S., Inema, I., Miller, S.E., Bieri, G., Zuchero, J.B., et al. (2014). Neuronal activity promotes oligodendrogenesis and adaptive myelination in the mammalian brain. Science *344*, 1252304.

Current Biology
Supplemental Information

CNS Myelin Sheath Lengths
Are an Intrinsic Property of Oligodendrocytes

Marie E. Bechler, Lauren Byrne, and Charles ffrench-Constant

Supplemental Data

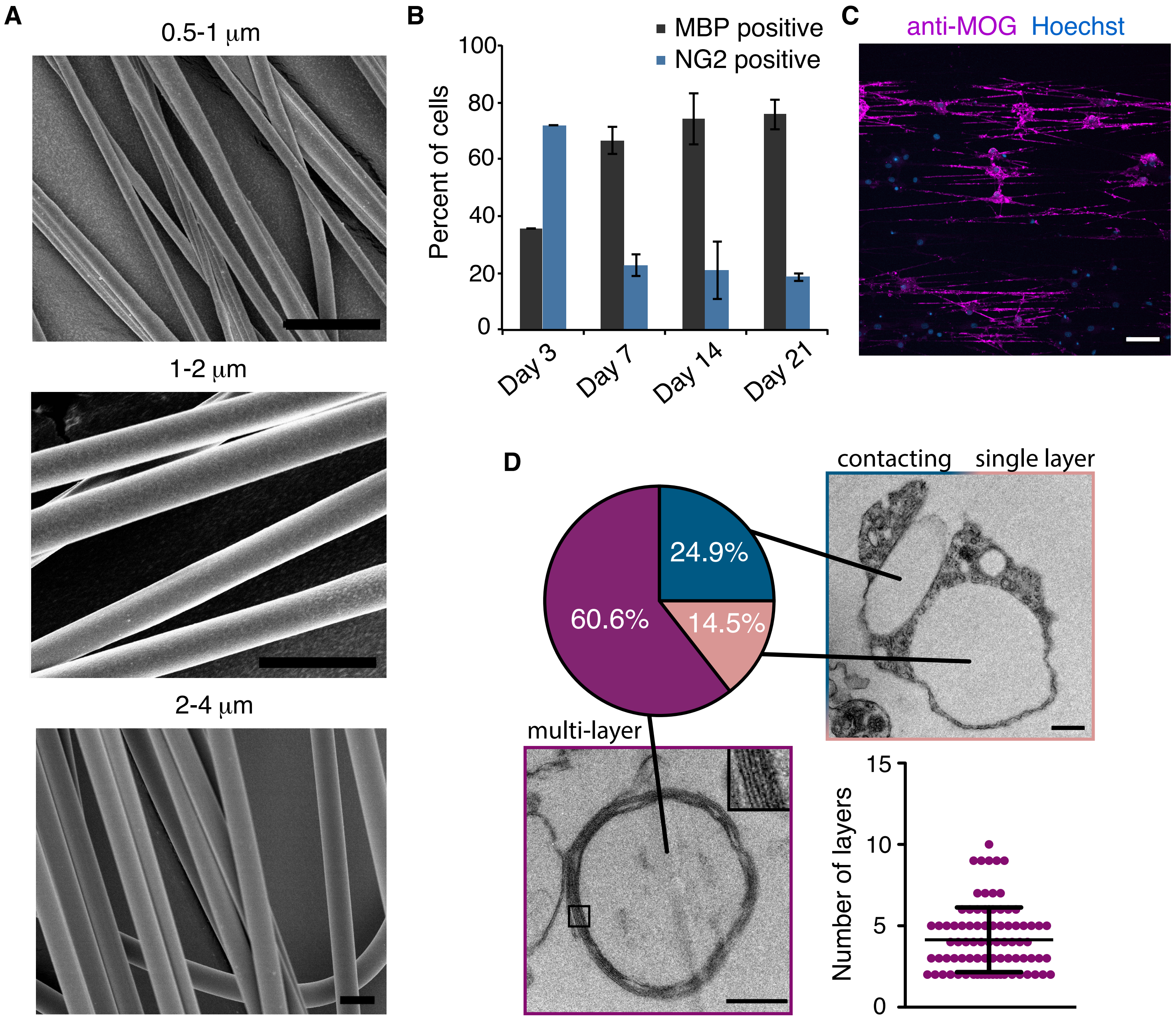

Figure S1. Oligodendrocyte precursors differentiate to mature oligodendrocytes on microfibers. Also see Figure 1. **(A)** Scanning electron micrographs of 0.5-1 µm, 1-2 µm, or 2-4 µm diameter fibers. Scale bars, 5 µm. **(B)** Percent of oligodendrocyte precursors (NG2) and oligodendrocytes (MBP) on 1-2 µm diameter microfibers. More than 600 cells were counted per condition. Mean and standard deviation are shown for three experiments, each with pooled cells from >3 animals. **(C)** Confocal stack shows that mature oligodendrocyte protein MOG is expressed and localized in sheaths. Scale bar, 40 µm. **(D)** Pie chart shows the percent of oligodendrocyte-fiber contact sites categorized as contacting only, single layer wrapping, and multi-layer wrapping. Example images of categories are shown. The number of layers of multilamellar wrapping is shown.

Table S1. Average sheath lengths with standard deviation.

The average log length and standard deviation were used for statistical tests, as the sheath length data was log normal (log lengths were Gaussian distributions). The equivalent mean sheath length in microns ± one standard deviation is shown (as lower and upper sheath length), calculated from the mean log(length) ± standard deviation of log(length).

Related figure	Condition	Mean log(length)	Standard deviation log(length)	Mean length (microns)	Lower length (microns)	Upper length (microns)
1 D,E	Day 21 neuron culture	1.300	0.022	20.0	19.0	21.0
1 D,E	Day 7 fiber culture	1.377	0.054	23.8	21.0	27.0
1 D,E	Day 14 fiber culture	1.370	0.100	23.4	18.6	29.5
1 D,E	Day 21 fiber culture	1.415	0.087	26.0	21.3	31.7

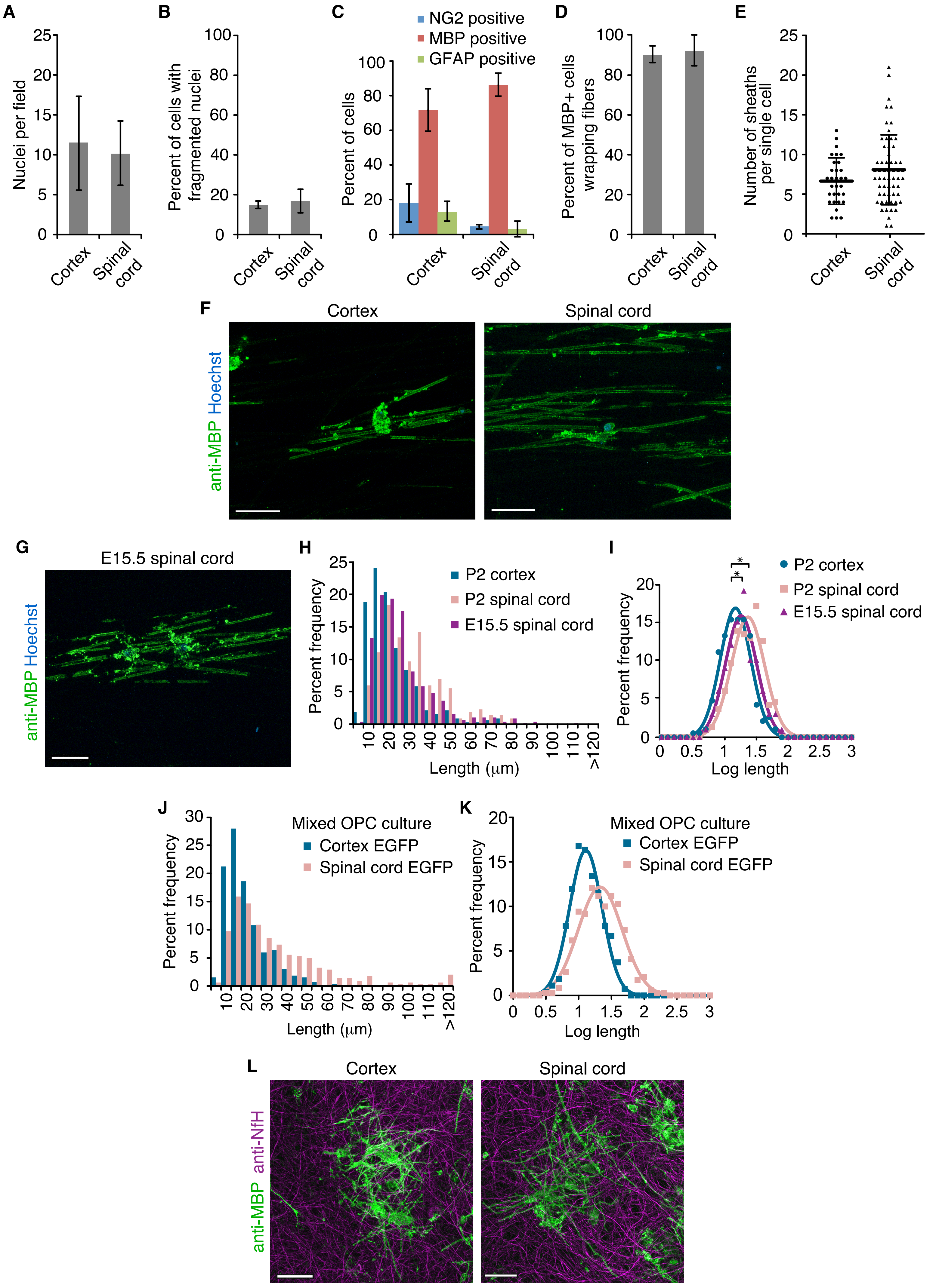

A
B
C
D
E
Nuclei per field
Percent of cells with fragmented nuclei
Percent of cells
NG2 positive
MBP positive
GFAP positive
Percent of MBP+ cells wrapping fibers
Number of sheaths per single cell
Cortex
Spinal cord
F
Cortex
Spinal cord
anti-MBP Hoechst
G
E15.5 spinal cord
anti-MBP Hoechst
H
Percent frequency
P2 cortex
P2 spinal cord
E15.5 spinal cord
Length (μm)
I
Percent frequency
P2 cortex
P2 spinal cord
E15.5 spinal cord
Log length
J
Percent frequency
Mixed OPC culture
Cortex EGFP
Spinal cord EGFP
Length (μm)
K
Percent frequency
Mixed OPC culture
Cortex EGFP
Spinal cord EGFP
Log length
L
Cortex
Spinal cord
anti-MBP anti-NfH

Figure S2. Oligodendrocytes have intrinsic regional identity that determines relative sheath lengths. Also see Figure 2. **(A)** The cell density of cortical and spinal cord oligodendrocytes is comparable after 14 days in culture on 1-2 μm microfibers, as seen by number of nuclei per image field. More than 500 cells were counted per condition. Mean and standard deviation are shown for five experiments, each with pooled cells from >3 animals. **(B)** A similar fraction of cells undergo cell death between cortical and spinal cord oligodendrocytes, as determined by fragmented nuclei after 14 days on 1-2 μm microfibers. More than 350 cells were counted per condition. Mean with standard deviation is shown for three experiments, each with pooled cells from >3 animals. **(C)** The percent of oligodendrocyte precursor cells (NG2 positive), differentiated oligodendrocytes (MBP positive), and astrocytes (GFAP positive) between cortex and spinal cord preparations is shown for Day 14 on 1-2 μm microfibers. More than 300 cells were counted per condition. Mean with standard deviation is shown for three experiments, each with pooled cells from >3 pooled animals. **(D)** The percent of MBP+ cells generating at least one sheath is shown for Day 14 spinal cord and cortical oligodendrocyte cultures on 1-2 μm fibers. **(E)** The number of sheaths formed by individual spinal cord or cortical oligodendrocytes is shown with mean and standard deviation for at least 31 cells from four experiments, each with pooled cells from >3 animals. **(F)** Confocal stacks of rat primary oligodendrocytes isolated from neonatal cortex or spinal cord, cultured 14 days on 1-2 μm microfibers. Scale bars, 40 μm. **(G)** Example of a confocal stacked image of E15.5 spinal cord oligodendrocytes on 1-2 μm microfibers cultured 14 days. Scale bar, 40 μm.
(H) Histogram of sheath lengths at Day 14 on 1-2 μm microfibers. More than 200 sheaths per condition were measured from 3 experiments, each with pooled cells from >3 animals. **(I)** Log sheath length frequency of neonatal cortical or spinal cord oligodendrocytes versus E15.5 spinal

cord oligodendrocytes is shown. Spinal cord oligodendrocyte (E15.5 or neonatal) mean log sheath lengths are significantly different than cortex, * $p < 0.02$, one-way ANOVA for mean log lengths. (**J**) Histogram showing sheath lengths from lentivirus-transduced, EGFP-expressing oligodendrocytes. EGFP-expressing spinal cord oligodendrocytes were mixed with unlabeled cortical oligodendrocytes, or EGFP-expressing cortical oligodendrocytes with unlabeled spinal cord oligodendrocytes, verifying that the sheath length differences are intrinsic to oligodendrocytes from these two regions. More than 250 sheaths were measured from two experiments, each with pooled cells from >6 pups added. **(K)** Frequency of log sheath lengths from indicated EGFP-expressing oligodendrocyte populations in mixed oligodendrocyte cultures. **(L)** Example confocal images of cortical and spinal cord oligodendrocytes added to cerebellar *shiverer* slice cultures. Scale bar, 40 µm.

Table S2. Average sheath lengths with standard deviation.

The average log length and standard deviation were used for statistical tests. The equivalent mean sheath length with lower and upper sheath lengths (mean + or – one standard deviation) is shown, calculated from the mean log(length) ± standard deviation of log(length).

Related figure	Condition	Mean log length	Standard deviation log length	Mean length (microns)	Lower length (microns)	Upper length (microns)
2 A,B	Cortex on fibers	1.091	0.150	12.3	8.7	17.4
2 A,B	Spinal cord on fibers	1.353	0.061	22.5	19.6	25.9
2 C,D	Cortex on neurons	1.403	0.100	25.3	20.1	31.8
2 C,D	Spinal cord on neurons	1.465	0.116	29.2	22.3	38.1
2 E,F	Cortex on slices	1.347	0.092	22.2	18.0	27.5
2 E,F	Spinal cord on slices	1.471	0.087	29.6	24.2	36.1
S2 G-I	P2 cortex	1.194	0.074	15.6	13.2	18.5
S2 G-I	P2 spinal cord	1.384	0.033	24.2	22.4	26.1
S2 G-I	E15.5 spinal cord	1.302	0.052	20.0	17.8	22.6
S2 J,K	Cortex EGFP	1.206	0.075	16.1	13.5	19.1
S2 J,K	Spinal cord EGFP	1.393	0.013	24.7	24.0	25.5

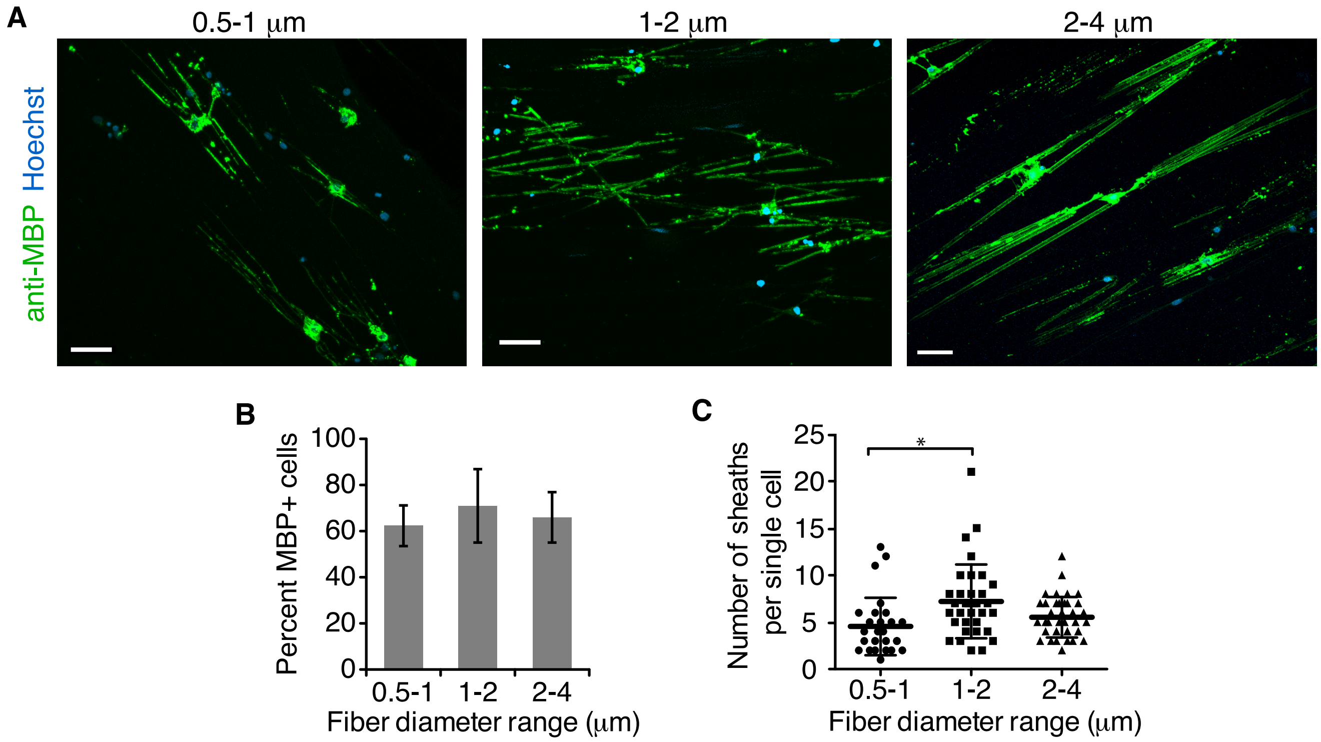

A
0.5-1 μm
1-2 μm
2-4 μm
anti-MBP Hoechst
B
Percent MBP+ cells
100
80
60
40
20
0
0.5-1
1-2
2-4
Fiber diameter range (μm)
C
Number of sheaths per single cell
25
20
15
10
5
0
*
0.5-1
1-2
2-4
Fiber diameter range (μm)

Figure S3. Oligodendrocytes have the ability to sense diameters to generate longer sheath lengths. Also see Figure 3. **(A)** Confocal stacks of oligodendrocytes cultured 14 days on varied diameter microfibers, as indicated. Scale bars, 40 μm. **(B)** The fraction of differentiated cells (MBP+) is not affected by microfiber diameter at Day 14. A minimum of 100 cells was counted for each diameter. Mean with standard deviation is shown from three experiments, each with pooled cells from >3 animals. **(C)** Sheath number formed by individual oligodendrocytes is shown with average and standard deviation indicated. At least 28 single cells were analyzed for each diameter from three experiments, each with pooled cells from >3 animals. Sheath number formed is slightly reduced on fiber diameters of less than one micron (* $p < 0.01$ Kruskal-Wallis, Dunn's multiple comparison test), but not significantly different on fiber diameters greater than one micron.

Table S3. Average sheath lengths with standard deviation.

The average log length and standard deviation were used for statistical tests. The equivalent mean sheath length with lower and upper sheath lengths (mean + or – one standard deviation) is shown, calculated from the mean log(length) ± standard deviation of log(length).

Related figure	Condition	Mean log length	Standard deviation log length	Mean length (microns)	Lower length (microns)	Upper length (microns)
3 A,C	Cortex, 0.5-1 μm diameter	0.893	0.118	7.8	6.0	10.2
3 A,C	Cortex, 1-2 μm diameter	1.091	0.150	12.3	8.7	17.4
3 A,C	Cortex, 2-4 μm diameter	1.447	0.093	28.0	22.6	34.7
3 B,C	Spinal cord, 0.5-1 μm diameter	1.099	0.074	12.6	10.6	14.9
3 B,C	Spinal cord, 1-2 μm diameter	1.353	0.061	22.5	19.6	25.9
3 B,C	Spinal cord, 2-4 μm diameter	1.744	0.054	55.5	49.0	62.7

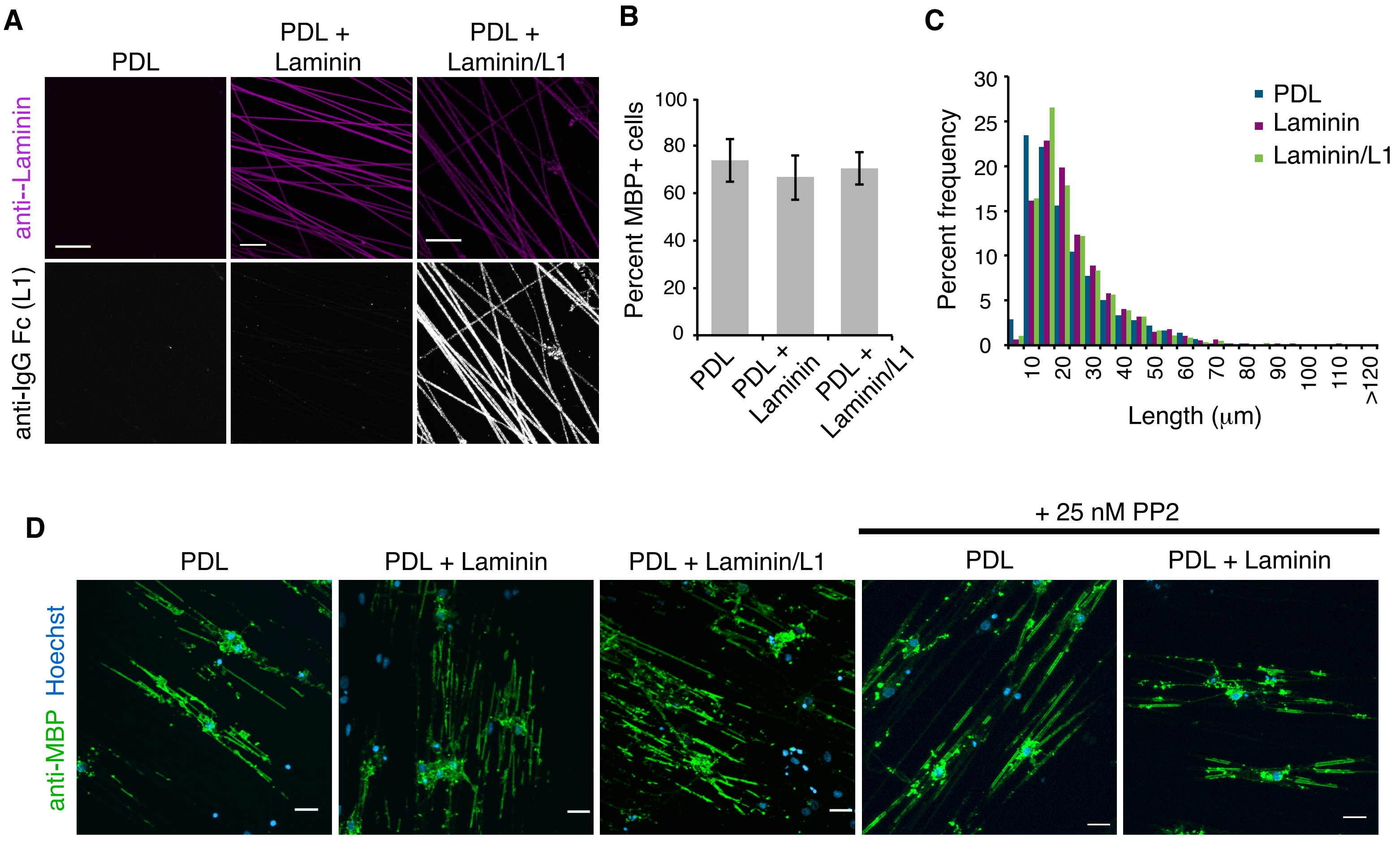

A
PDL
PDL + Laminin
PDL + Laminin/L1
anti--Laminin
anti-IgG Fc (L1)
B
Percent MBP+ cells
100
80
60
40
20
0
PDL
PDL + Laminin
PDL + Laminin/L1
C
Percent frequency
30
25
20
15
10
5
0
PDL
Laminin
Laminin/L1
10
20
30
40
50
60
70
80
90
100
110
>120
Length (μm)
D
+ 25 nM PP2
PDL
PDL + Laminin
PDL + Laminin/L1
PDL
PDL + Laminin
anti-MBP Hoechst

Figure S4. The number of sheaths produced by cortical oligodendrocytes but not sheath lengths is increased on laminin-coated microfibers. Also see Figure 4. **(A)** Confocal images verifying microfiber coating with laminin (anti-laminin) and L1 (anti-IgG Fc) recombinant proteins. Scale bars, 40 µm. (**B)** The percent of differentiated (MBP+) cortical oligodendrocytes is not changed in the presence of laminin or laminin+L1 coating. **(C)** Histogram of cortical oligodendrocyte sheath lengths. More than 900 sheaths were measured per condition from 3 experiments, each with pooled cells from >3 animals added. **(D)** Confocal images of cortical oligodendrocytes cultured 14 days on 1-2 µm microfibers coated as indicated ± addition of the Fyn inhibitor PP2. Scale bars, 20 µm.

Table S4. Average sheath lengths with standard deviation.

The mean log length and standard deviation were used for statistical tests. The equivalent mean sheath length with lower and upper sheath lengths (mean + or – one standard deviation) is shown, calculated from the mean log(length) ± standard deviation of log(length).

Related figure	Condition	Mean log length	Standard deviation log length	Mean length (microns)	Lower length (microns)	Upper length (microns)
4 B, S4 C	PDL	1.196	0.077	15.7	13.2	18.7
4 B, S4 C	PDL + Laminin	1.196	0.097	15.7	12.6	19.6
4 B, S4 C	PDL + Laminin/L1	1.223	0.021	16.7	15.9	17.5

Supplemental Experimental Procedures

Primary Cell Culture

All primary cell isolation was done in accordance with regulations of the Animal (Scientific Procedures) Act under an issued UK Home Office project license. Oligodendrocyte precursor cells were isolated from pooled P0-P2 Sprague-Dawley neonatal rat cerebral cortices as well as P0-P2 or E15.5 spinal cords from one or more litters. Cortices and spinal cords were isolated, meninges removed, minced, and cells were dissociated for 60 min at 37°C with 1.2 U/mL papain (Worthington), 0.1 mg/mL L-cysteine (Sigma-Aldrich) and 0.40 mg/mL DNase I (Sigma-Aldrich). The resulting mixed glial cultures were plated onto poly-D-lysine (PDL) coated flasks and cultured at 37°C in 7.5% CO_2 in DMEM (Invitrogen 41966029), 10% fetal bovine serum (FBS, Invitrogen) and 1% penicillin/streptomycin (pen/strep, Invitrogen). Mixed glial cultures were separated after 10 days by mechanical shaking at 240 rpm for 1 h to remove microglia followed by additional 18 h shaking [S1]. Cells collected were plated onto petri dishes 20 min for differential adhesion to further remove microglia and astrocytes. The enriched oligodendrocyte precursor cells (OPCs) were collected and plated at 35,000 onto fibers or 70,000 onto DRG neurons in myelin media: 50:50 DMEM:Neurobasal Media, B27 (Invitrogen), 5 µg/mL N-acetyl cysteine, and 10 ng/mL D-biotin, ITS , and modified Sato (100 µg/mL BSA fraction V, 60 ng/ml Progesterone, 16 µg/ml Putrecsine, 400 ng/mL Tri-iodothyroxine, 400 ng/mL L-Thyroxine; reagents from Sigma-Aldrich).

Dorsal root ganglia neurons (DRGs) were isolated and cultured as previously described [S2]. DRGs were removed from a litter of E15.5 Sprague Dawley rat embryos and dissociated with 1.2 U/mL papain, 0.24 mg/mL L-cysteine and 0.40 mg/mL DNase I for 60 min at 37°C. Cells were

plated at 150,000 cells per 18 mm coverslip, coated with PDL and growth factor-reduced Matrigel (BD Biosciences). DRG neurons were cultured at 37°C, 7.5% CO_2 in DMEM with 10% FBS, 1% pen/strep, and 100 ng/mL nerve growth factor (NGF, AbD Serotec). To reduce contaminating cell growth, cultures were pulse-treated 3 times with 10 μM 5-fluoro-2'-deoxyuridine (Sigma-Aldrich) for 3 days each on/off, starting within 16 h after plating. After 21 days, the media was changed to the appropriate myelination media.

Primary rat Schwann cells were obtained from pooled P0-P2 neonatal Sprague-Dawley rat sciatic nerves. Sciatic nerves were dissociated with 1 mg/mL Collagenase II (Invitrogen 17101-015) for 30 min at 37°C, followed by Collagenase II/Trypsin dissociation at 37°C for 30 min. Cells were immunopanned twice using an OX7 supernatant (provided generously by the Brophy lab, The University of Edinburgh, UK)-coated plate before culturing on a PDL-coated plate at 37°C 7.5% CO_2 in DMEM, 10% FBS, 1% pen/strep, 10 ng/mL Neuregulin1 (Nrg1, R&D Systems 396-HB), and 2 μg/mL forskolin (Sigma-Aldrich F6886). Schwann cells were maintained for a maximum of three passages after plating, and used at 35,000 or 70,000 cells per fiber or DRG co-culture, respectively. For myelination, Schwann cells were cultured in DMEM/F-12 (Invitrogen 11320033) supplemented with Sato, ITS, pen/strep, 2% FBS, and 50 μg/mL freshly prepared ascorbic acid.

For labelled cell experiments, 250,000 oligodendrocyte precursor cells were plated into each well of a 6-well plate with DMEM, ITS, modified Sato's medium with 0.5% FBS, 10 ng/ml PDGF, and 10 ng/ml FGF for a day. After 24 h cells were incubated overnight with lentivirus at 10 MOI for EGFP expression. Lentiviruses were obtained from the Biomolecular Core Facility in the

MRC Human Reproductive Sciences Unit (The University of Edinburgh, UK). Cells were collected by TrypLe (Invitrogen) detachment and counted. EGFP labelled cells were mixed equally with unlabeled cells (i.e. EGFP expressing cortical OPCs with unlabeled spinal cord OPCs, and vice versa). Cells were then cultured on fibers as above.

Slice Cultures

The cerebellum of a P3 *shiverer* mouse (*Mbp*^{shi}/*Mbp*^{shi} from the C3Fe.SWV-*Mbp*^{shi}/J genetic background) was sectioned sagitally in 300 µm slices on a McIlwain tissue chopper, and plated onto Millipore-Millicel-CM mesh inserts in 6-well culture plates. Media was composed of 50% minimal essential media, 25% heat-inactivated horse serum, 25% Earle's balanced salt solution, 6.5 mg/ml glucose (Sigma), 1% pen/strep, and Glutamax (Invitrogen), and was changed every 2-3 days. After 2 days in culture, 50,000 EGFP-expressing cortical or spinal cord oligodendrocytes were added. Fourteen day after addition of OPCs, slices were fixed 1 h in 4% PFA in PBS, washed 3 x 1 h in PBS, blocked for 1 h. Primary antibodies were incubated for 36 h at 4°C, slices were washed, and fluorescently conjugated antibody was applied overnight at 4°C. Slices were mounted onto glass slides using Fluoromount-G (Southern Biotech).

Electrospun Fibers

Custom parallel-aligned electrospun fibers composed of poly-L-lactic acid were synthesized and suspended by fitting into 12-well plate inserts by The Electrospinning Company. Fiber diameter ranges that are known to promote myelin sheath formation in vivo were used [S3], at densities that produced < 10 µm spacing. Fiber diameter ranges of 0.5-1 µm, 1-2 µm, and 2-4 µm were

generated and were verified with scanning electron microscopy (SEM, Figure S1A). Prior to use, inserts were soaked in 70% ethanol, washed, coated 1 h at 37°C with 5 μg/mL PDL, followed by three washes to remove excess PDL. For laminin experiments, fibers were further coated by a solution of 10 μg/mL human placental laminin (Sigma, L6274) with or without 5 μg/mL L1 (R&D Systems, 777-NC) overnight at room temperature, followed by three washes.

Immunofluorescence

Cells were washed once with phosphate buffered saline, pH 7.4 (PBS), fixed in 4% paraformaldehyde/PBS, followed by PBS washes, and permeabilization in 0.1% TritonX-100 in PBS. Primary antibodies were diluted in PBS, as indicated below, and incubated overnight at 4°C. Primary antibodies used were: rat anti-myelin basic protein (MBP) 1:250 (AbD Serotec), chicken anti-neurofilament (NfH) at 1:1000 (Sigma-Aldrich), rabbit or mouse anti-NG2 1:200 or 1:100 (Millipore), mouse anti-MOG 1:100 (Millipore), rabbit anti-S100 1:250 (Dako), rabbit anti-GFAP 1:500 (Dako), goat anti-human IgF Fc 1:250 (Jackson Immunoresearch), chicken anti-GFP 1:500 (Abcam), and mouse anti-laminin 1:100 (Millipore). Cells were washed in PBS again and incubated 1 h with Alexafluor 488, 568, or 647 conjugated secondary antibodies against rat, rabbit, mouse, goat, or chicken (Invitrogen), used at a 1:1000 dilution. After PBS washes, cells were stained with 5 μg/mL Hoechst (Sigma-Aldrich), washed again and mounted with Fluoromount G (Southern Biotech) with cover glass onto glass slides.

Fluorescence Imaging and Image Analysis

Confocal images were obtained on a Leica SPE confocal or SP8 confocal scanning microscope, with 20x/NA0.5, 40x oil/NA1.3, and 40x oil/NA1.25 objectives. Confocal stacks of 0.38-0.5 μm z-steps were taken as necessary at 1024x1024. At least 10 random areas across each coverslip were imaged. The same settings (gain, binning, z-steps, etc.) were used between coverslips in a single experiment. Any brightness or contrast adjustments were done uniformly across all images from a single experiment.

The number of MBP-positive cells, in combination with Hoechst nuclei staining, was counted with at least 80 cells counted per coverslip per experiment. Similarly, NG2 or GFAP positive cells were counted. The percent of wrapping MBP+ cells was determined by counting the number of MBP+ cells that were forming at least one sheath surrounding fibers. For sheath measurements, a sheath was defined as a continuous, smooth tube of MBP staining surrounding an axon or fiber (Figure 1B). For all experiments, except those comparing fiber diameters, lengths were measured from blinded confocal images by drawing and acquiring length measurements in Fiji [S4] version of ImageJ (NIH). For initial experiments comparing spinal cord and cortical oligodendrocytes, experiments were reproduced by two individuals. For experiments comparing fiber diameters, however, it was not possible to blind experiments, as the diameters were evident upon viewing the images. Independent experiments (i.e. cells pooled from different rat litters on different days) were conducted with a minimum of 200 sheaths measured for microfiber experiments (as indicated in figure legends). Statistical analysis was done in GraphPad Prism 5. For differentiation analysis, one-way ANOVAs were conducted. For comparison of multiple length distributions, the Gaussian distribution of log lengths was

used to compare mean log lengths between experiments by one-way ANOVA with Tukey's multiple comparison post-testing. For comparison of two length distributions (spinal cord versus cortical oligodendrocytes), a paired two-tailed t-test was used for mean log lengths. For single cell analysis (sheath number per single cell), the number of sheaths for each individual MBP-positive cell was measured and counted. A normal distribution was not assumed for sheath number; therefore a Kruskall-Wallis test with Dunn's multiple comparison post-testing was used.

Electron Microscopy

Cells were briefly washed in 0.1 M phosphate buffer, pH 7.2 (PB), followed by 2 h fixation in 2.5% glutaraldehyde/4% paraformaldehyde in PB. Cells were washed in PB then fixed 1 h at 4°C with 1% Osmium tetroxide (Electron Microscopy Sciences) in PB. Cells were rinsed further in PB, and then dehydrated with a gradient of 50, 75, 90, and 100% ethanol 2 times each. For transmission electron microscopy, after dehydration, samples were placed into 50:50 mix of resin:ethanol for an hour, followed by multiple resin changes over 48 h. Resin was either Agar100 or LR White (Agar Scientific). Resin was then changed and cured for a minimum of 24 h at 60°C. Ultrathin 60 nm sections were cut, followed by Uranyl Acetate and Lead Citrate post-staining (Electron Microscopy lab, Institute of Molecular Plant Sciences, The University of Edinburgh, UK). Images were acquired on a Phillips CM120 transmission electron microscope equipped with a Gatan Orius CCD camera. For scanning electron microscopy, samples were critical point dried post-ethanol dehydration, and mounted on aluminum stubs for palladium/gold sputter coating. SEM images were acquired on a XL30CP scanning electron microscope (Philips). The number of contacting, wrapping (single layer), and multilayer wrapping occurrences was counted from images of 1-2 micron fibers wherever an oligodendrocyte process

was in contact with fibers (contact with cell bodies were excluded). A total of 350 across 7 biological replicates (different cell isolations on different days) were counted to determine percent for each category.

Supplemental References

S1. McCarthy, K. D., and de Vellis, J. (1980). Preparation of separate astroglial and oligodendroglial cell cultures from rat cerebral tissue. J. Cell. Biol. *85*, 890–902.

S2. Laursen, L. S., Chan, C. W., and ffrench-Constant, C. (2009). An integrin-contactin complex regulates CNS myelination by differential Fyn phosphorylation. J. Neurosci. *29*, 9174–9185.

S3. Hildebrand, C., Remahl, S., Persson, H., and Bjartmar, C. (1993). Myelinated nerve fibres in the CNS. Prog. Neurobiol. *40*, 319–384.

S4. Schindelin, J., Arganda-Carreras, I., Frise, E., Kaynig, V., Longair, M., Pietzsch, T., Preibisch, S., Rueden, C., Saalfeld, S., Schmid, B., et al. (2012). Fiji: an open-source platform for biological-image analysis. Nat. Meth. *9*, 676–682.
